# Crk adaptor proteins act as key signaling integrators for breast tumorigenesis

**DOI:** 10.1186/bcr3183

**Published:** 2012-05-08

**Authors:** Kelly E Fathers, Emily S Bell, Charles V Rajadurai, Sean Cory, Hong Zhao, Anna Mourskaia, Dongmei Zuo, Jason Madore, Anie Monast, Anne-Marie Mes-Masson, Andree-Anne Grosset, Louis Gaboury, Michael Hallet, Peter Siegel, Morag Park

**Affiliations:** 1 Department of Biochemistry, McGill University, Rosalind and Morris Goodman Cancer Research Centre, 3655 Promenade Sir William Osler, Montréal, QC H3G 1Y6, Canada; 2 Department of Bioinformatics, McGill University, Rosalind and Morris Goodman Cancer Research Centre, 3655 Promenade Sir William Osler, Montréal, QC H3G 1Y6, Canada; 3 Department of Oncology, McGill University, Rosalind and Morris Goodman Cancer Research Centre, 3655 Promenade Sir William Osler, Montréal, QC H3G 1Y6, Canada; 4 Departmet of Medicine, McGill University, Rosalind and Morris Goodman Cancer Research Centre, 3655 Promenade Sir William Osler, Montréal, QC H3G 1Y6, Canada; 5 Centre de recherche du Centre hospitalier de l'Université de Montréal, Institut du cancer de Montréal, 1560 rue Sherbrooke est, Montreal, QC H2L 4M1, Canada; 6 Département de Médicine, Université de Montréal, 2900 boulevard Édouard-Montpetit, Montreal, QC H3T 1J4, Canada; 7 IRIC-Institut de Recherche en Immunologie et Cancerologie, Université de Montréal, 2950 chemin de Polytechnique, Montreal, QC H3T 1J4, Canada; 8 Department of Cell Biology, The Hospital for Sick Children, Toronto Medical Discovery Tower, 101 College Street, Toronto, ON M5G 1X8, Canada

## Abstract

**Introduction:**

CT10 regulator of kinase (Crk) adaptor proteins (CrkI, CrkII and CrkL) play a role in integrating signals for migration and invasion of highly malignant breast cancer cell lines. This has important implications, as elevated CrkI/II protein levels were observed in a small cohort of breast cancer patients, which identified a potential role for Crk proteins in breast cancer progression. Numerous *in vitro *studies identified a role for Crk proteins in cell motility, but little is known about how Crk proteins contribute to breast cancer progression *in vivo*.

**Methods:**

The clinical significance of Crk proteins in human breast cancer was assessed by analyzing published breast cancer datasets using a gene expression signature that was generated following CrkII over-expression and by examining Crk protein expression in tissue microarrays of breast tumors (*n *= 254). Stable knockdown of Crk (CrkI/CrkII/CrkL) proteins was accomplished using a short hairpin RNA (shRNA)-mediated approach in two basal breast cancer cell lines, MDA-231 1833TR and SUM1315, where the former have a high affinity to form bone metastases. Both *in vitro *assays (cell migration, invasion, soft agar growth) and *in vivo *experiments (intra-cardiac, tibial and mammary fat pad injections) were performed to assess the functional significance of Crk proteins in breast cancer.

**Results:**

A gene signature derived following CrkII over-expression correlated significantly with basal breast cancers and with high grade and poor outcome in general. Moreover, elevated Crk immunostaining on tissue microarrays revealed a significant association with highly proliferative tumors within the basal subtype. RNAi-mediated knockdown of all three Crk proteins in metastatic basal breast cancer cells established a continued requirement for Crk in cell migration and invasion *in vitro *and metastatic growth *in vivo*. Furthermore, Crk ablation suppressed anchorage independent growth and *in vivo *orthotopic tumor growth. This was associated with diminished cell proliferation and was rescued by expression of non-shRNA targeted CrkI/II. Perturbations in tumor progression correlated with altered integrin signaling, including decreased cell spreading, diminished p130Cas phosphorylation, and Cdc42 activation.

**Conclusions:**

These data highlight the physiological importance of Crk proteins in regulating growth of aggressive basal breast cancer cells and identify Crk-dependent signaling networks as promising therapeutic targets.

## Introduction

Crk, or CT10 regulator of kinase, was originally isolated as the oncogene fusion product of the CT10 chicken retrovirus (v-Crk) [1]. Cellular homologues of v-Crk include the c-Crk gene, which encodes two alternatively spliced mRNAs that give rise to two proteins (c-CrkI and c-CrkII) and a second gene, c-CrkL [2]. Crk adaptor proteins contain Src homology 2 (SH2) and Src homology 3 (SH3) domains, which mediate the formation of protein-protein complexes. As adaptor proteins, Crk proteins are considered highly pleiotropic, as they have been proposed to regulate cell migration, invasion, and survival downstream of integrins and various receptor tyrosine kinases [3]. For instance, in response to hepatocyte growth factor, Crk adaptor proteins are required for the dispersal of organized epithelial colonies, formation of lamellipodia and the breakdown of adherens junctions, events that are critical to tumor cell dispersal and, hence, cancer progression [4]. Furthermore, over-expression of Crk promotes an invasive phenotype regardless of upstream signaling, which identifies Crk as a potential key regulator of cell invasion [5]. In support of this, transient knockdown of CrkI/II resulted in significant inhibition of migration and invasion of multiple malignant breast (MDA-231, MDA-435s) and other human cancer cell lines (HeLa, H1299, KB) [6], confirming that CrkI/II are critical integrators of upstream signals for cell migration and invasion and highlighting a potential role for these adaptor proteins in metastatic spread.

Elevated expression of Crk proteins has also been linked to cell transformation, with CrkI exhibiting the highest transforming potential. CrkI, CrkII and CrkL over-expressing fibroblasts proliferate in soft agar, although only CrkI over-expressing fibroblasts form tumors in nude mice [2,7,8]. However, a mouse model over-expressing CrkL exhibited an increased incidence of hematopoietic and epithelial cancers and a mammary mouse model over-expressing CrkII was associated with altered mammary gland development and accelerated tumor development [9,10]. Although tumor incidence was low in the latter study, the ability of CrkII to induce a branching phenotype during a normally quiescent state suggests that CrkII may play an active role in mammary epithelial proliferation and remodeling [9]. This precocious development, coupled with the fact that CrkII is downstream of several signaling proteins involved in breast cancer development, highlights the potential consequence of elevated Crk proteins in the human disease.

Elevated levels of CrkI and CrkII mRNA and protein are found in various human tumors, including glioblastoma [11], gastric and breast cancers [6,12,13]. CrkI/II mRNA expression was increased predominantly in advanced lung tumors and those associated with poor survival, implying that Crk proteins may play a key role in epithelial human cancers [14].

The complexity and multi-step nature of tumor initiation, maintenance and metastasis has hindered the elucidation of key molecular processes defining breast cancer progression. Although Crk proteins have been characterized in the context of cellular signaling, particularly through over-expression studies, and demonstrate aberrant expression in multiple cancer types, including breast, few studies have addressed the precise role of Crk proteins in breast cancer-related processes *in vivo*. Since Crk is proposed to be involved in many aspects of tumorigenesis, dissecting whether Crk is required in tumor initiation and progression is an important step towards developing targeted therapeutics for breast cancer. Here, we demonstrate that elevated Crk is significantly associated with highly proliferative breast tumors of triple negative subtype and a gene expression signature derived following CrkII over-expression correlated with basal breast cancer, implicating Crk signaling in an aggressive breast cancer phenotype. To confirm the functional significance of Crk proteins within basal breast cancer, we demonstrate that Crk proteins are critical for the growth of breast cancer cells *in vivo *at both orthotopic and metastatic sites, highlighting the physiological importance of Crk proteins in regulating cancer signaling. This data implicates Crk proteins not only in the enhanced malignancy of breast cancer, but also in an aggressive breast cancer phenotype.

## Methods

### Reagents, cell lines and retroviral transduction

The MDA-231 1833TR cell line was generously provided by Joan Massagué in 2004. This cell line and its triple reporter (TR) were previously described [15,16] and cells used in this study were expanded from freeze downs from 2004. The SUM1315 cell line was purchased from Asterand (Detroit, MI, USA) in 2009 and cultured as in [17] from freeze downs from 2009. Both cell lines underwent standard mouse antibody production (MAP) testing for pathogens, as well as mycoplasm testing. CrkL short hairpin RNA (shRNA) (V2HS_43900, Open Biosystems, Huntsville, AL, USA) was subcloned into LMP according to [18]. pSuperCrkI/II constructs were made according to the manufacturer's protocol. The CrkI/II shRNA sequence was based on the duplex published in [6]. Retrovirally-generated LMP or LMP-CrkL shRNA cell lines were subsequently infected with pSuper or pSuperCrkI/II. CrkI/II rescue cell lines were made using pLXSP-CrkI and pLXSH-CrkII constructs containing silent mutations in the shRNA-targeted region from site directed mutagenesis using the QuickChange Multi Site-Directed Mutagenesis Kit from Stratagene (La Jolla, CA, USA). Antibodies included: CrkI/II, Rac1 and p130Cas (BD Transduction Laboratories, Mississauga, ON, Canada), CrkII (Novus Biologicals, Oakville, ON, Canada), CrkL, Cdc42 and actin (Santa Cruz Biotechnology, Santa Cruz, CA, USA), phospho-p130Cas (Cell Signaling Technology, Mississauga, ON, Canada), paxillin and alpha-tubulin (Sigma-Aldrich, Oakville, ON, Canada), and AlexaFluor 488 phalloidin (Molecular Probes, Eugene, OR, USA).

### Cellular assays

Cell spreading, migration and invasion assays are described in [6]. Analysis was performed with ImageScope (Aperio, Vista, CA, USA). A minimum of 300 cells were counted per condition for cell spreading. For soft agar assays, cells were plated as in [19], except grown in 5% serum and quantified using Infinity Analyze Software (Lumenera Corp., Ottawa, ON, Canada). Immunohistochemistry and immunofluorescence were performed according to [6,9]. Human breast tumor sections for immunofluorescence were obtained courtesy of the Breast Cancer Functional Genomics Group at McGill University.

### *In vivo *experiments

Female nude mice (six to eight weeks old, Charles Rivers) were injected with 10^6 ^cells into the mammary fat pad; tumor volumes were monitored bi-weekly and calculated as described [20]. Cardiac injections were performed using 10^5 ^cells as described [15]. Mice were sacrificed six to eight weeks post-injection. Tibial injections were performed using 10^4 ^cells injected directly into the marrow space. Ten mice were used per cell line and were sacrificed four weeks post-injection. Tumor outgrowth within bone was monitored according to [15]. Osteolytic lesions were blindly scored using X-ray radiographs. Total tumor area versus total tumor/bone interface was scored using ImageJ. All animal experiments were carried out in accordance with the guidelines of the McGill University Animal Ethics Committee and the Canadian Council on Animal Care as a protocol approved by the Facility Animal Care Committee (Protocols #4170 and #5562). The human tumor samples used in this study were collected from breast cancer patients at time of surgery. Tissue collection was conducted under protocols approved by the McGill University Health Centre (MUHC) Research Ethics Board (Protocols SUR-99-780 and SUR-00-966). All subjects provided written, informed consent.

### Western blot analysis

Cells were serum starved for four hours, plated on collagen for 30 minutes and protein lysates were resolved by SDS-PAGE [6]. Detection of GTP-bound endogenous Rac1 or Cdc42 was performed according to [21]. Densitometry was performed using ImageJ or Odyssey (LI-COR, Guelph, ON Canada).

### Crk signature and microarray profiling

A Crk signature was derived from expression analysis of T47D-CrkII cell lines [9] by selecting genes significantly differentially expressed between T47D and T47D-CrkII cell lines (FDR corrected *P*-value < 0.05 and fold change > 2, GSE ID: GSE30731). Expression of this signature was examined in breast cancer datasets and a breast cancer cell line dataset [22-27]. Molecular subtypes were determined using PAM50 centroids [23]. Patients were ordered based on correlation with the expression of the CRK signature in tumor samples. The association between patient ordering, molecular subtype and grade was determined using a Krusal-Wallis rank sum test. Analysis was conducted using Bioconductor [28].

### Tissue microarrays

Tissue microarray 1 (TMA-1) consisted of 113 patients diagnosed with breast cancer at the Centre Hospitalier de l'Universite de Montreal, Hôtel Dieu, and Notre Dame and consisted of low grade (Grade I, *n *= 15) and high grade (Grade III, *n *= 74) ductal carcinomas and typical (*n *= 9) and atypical (*n *= 15) medullary carcinomas [29], approved by the research ethics committee (Centre Hospitalier de l'Université de Montréal). TMA-2 consisted of 141 cases selected from consenting patients who had undergone surgical resection for breast cancer at the Centre Hospitalier de l'Université de Montréal, Hôtel Dieu. Representative tumor areas were annotated by a pathologist, and two single core TMAs were constructed in parallel. The TMA-2 clinical parameters included samples from grade 1 (*n *= 17), grade 2 (*n *= 39), and grade 3 (*n *= 83) patients. Tumors were of ductal (*n *= 102), lobular (*n *= 20) or other (*n *= 19) morphology. Immunohistochemistry for HER2, ER and PR identified 29 triple negative breast tumors. Crk positivity and the percentage of Ki67 positive cells were assessed using ImageScope. Crk staining intensity was also reported by a pathologist and an independent observer.

### Statistical analysis

A two-sample variance student's t-test was used to assess for statistical significance. Kendall's tau non-parametric statistical analysis was used to assess significance between Crk expression and Ki67 within TMA-1 and TMA-2.

## Results

### A CrkII-associated transcriptional signature and Crk protein expression correlate with a proliferative basal breast cancer subtype

**Figure 1:**
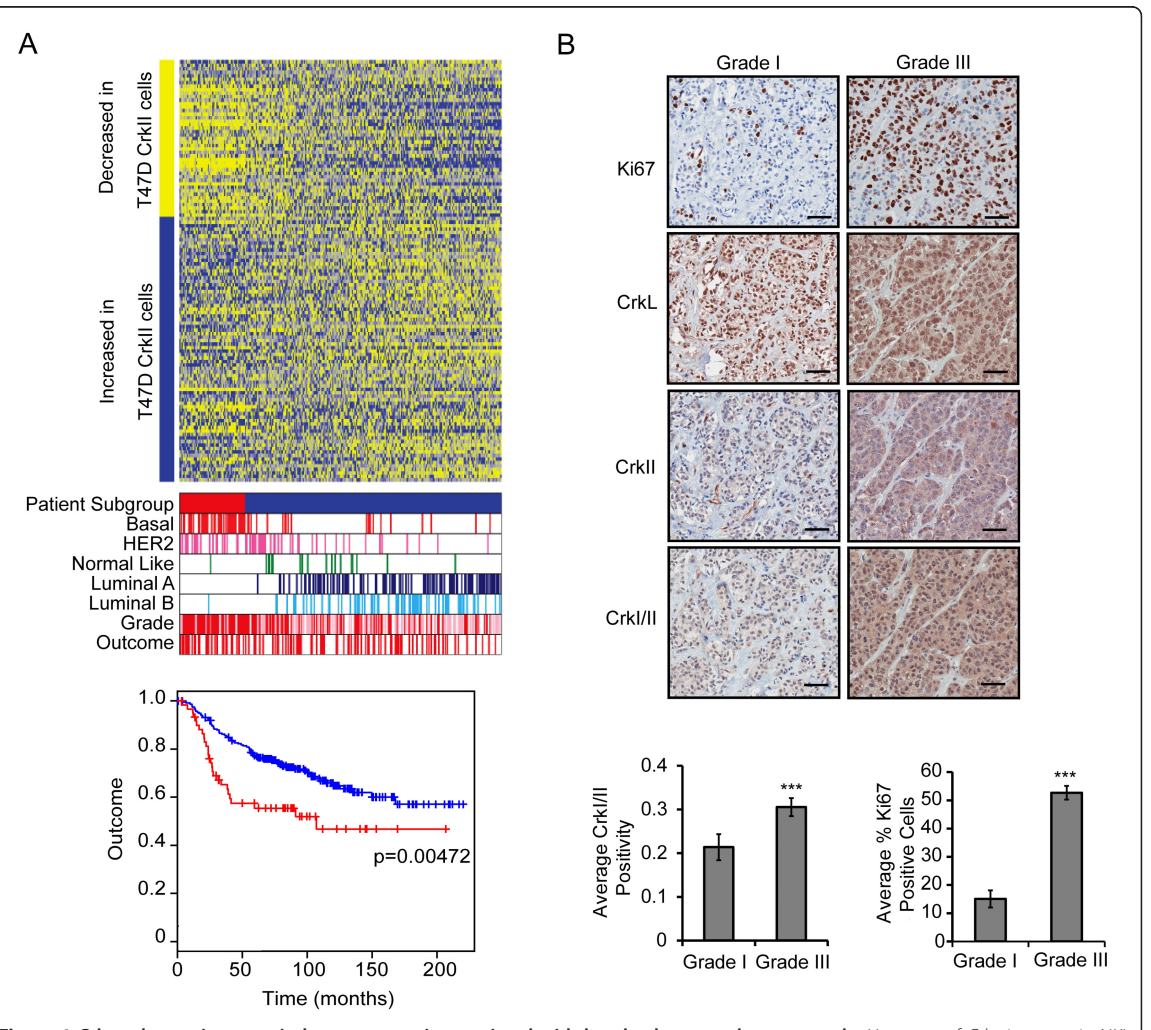
**Crk pathway signature in breast cancer is associated with basal subtype and tumor grade**. Heatmap of Crk signature in NKI breast cancer dataset. Bars on the left indicate if genes increased or decreased expression in the T47D-CrkII cell line compared to control. The patients are ordered by correlation of the expression of these 151 genes in the patients sample to the Crk signature. Patients were separated into two subgroups: high correlation (correlation > 0.17, red) and low correlation (correlation < 0.17, blue). On heatmap, blue indicates increased expression; yellow indicates decreased expression. The Crk signature was used to examine overall 20 year outcome; the red line indicates patients who exhibit the Crk gene signature, whereas the blue line represents patients whose tumors do not express the signature (**A**). TMA-1 was stained with Ki67, CrkI/II, CrkII or CrkL specific antibodies in high (Grade 3) and low (Grade 1) grade tumors. Images taken using ImageScope; scale bar represents 50 μm. TMA-1 was utilized to examine CrkI/II and Ki67 expression in relation to tumor grade. Errors bars represent standard error of the mean (SEM) (**B**). Statistically significant data is illustrated as (* = *P *< 0.05, ** = *P *< 0.01, *** = *P *< 0.001).

Crk over-expression has been linked to various aspects of tumorigenesis, including cell migration, invasion and anchorage independent growth [2,7,30]. Although elevated CrkI/II proteins have been reported in a small cohort (*n *= 20) of breast cancer samples [6], no studies have addressed if elevated Crk, or its signaling pathways, are associated with known clinical variables or breast cancer subtype. Gene expression profiling is a valuable tool to search for links between signaling networks and defined steps of tumor progression. Thus, to investigate an association between Crk-mediated signaling with breast cancer outcome, we constructed a gene expression signature composed of 151 genes that are most differentially expressed following CrkII over-expression (Additional file 1) [9]. This CrkII transcriptional signature was derived following gene expression profiling of the breast cancer cell line T47D versus T47D cells over-expressing CrkII [9]. Expression of 'Crk signature' genes was examined in five independent datasets (Figure 1a, Additional file 2) for a total of 1,469 breast cancer patients [22-26]. In each dataset, patients were ordered by expression of Crk signature genes (Additional file 1). Notably, the 'Crk gene signature' strongly correlated with the basal molecular subtype (*P *< 6.7e-14) in the NKI dataset (Figure 1a) [31]. In addition, there was a significant correlation with the basal subtype within the four additional breast cancer datasets examined (Ivshina, Loi, Parker and Wang, Additional file 2). The Crk signature also significantly associated with high tumor grade (*P *< 6.4e-12) and poor outcome (*P *= 0.00472) within the NKI (Figure 1a) and the additional four breast cancer datasets examined (Additional file 2). Thus, a CrkII-dependent gene signature is associated with high grade, basal breast cancers and poor outcome. The T47D cancer cell line used to generate the Crk gene signature is identified as a luminal subtype [27], thus, a dataset derived from a panel of breast cancer cell lines [27] was examined to confirm that the Crk signature is indeed more common within the basal subtype. Similar to the data obtained using datasets derived from human breast cancer patients, the Crk signature more closely correlated with the two basal subtypes (Basal A and Basal B) typically found within a subset of breast cancer cell lines (Additional file 3). Since an expression signature associated with high grade basal tumors and cell lines could be generated from the luminal T47D cell line over-expressing CrkII, it provides evidence for a role for Crk in promoting key signaling events in the progression of basal breast cancer.

To confirm whether elevated Crk proteins are associated with a breast cancer subtype or outcome, we also performed immunohistochemical staining using various Crk antibodies (CrkI/II, CrkII, CrkL C-20, CrkL H-62) on two independent human breast cancer tissue TMAs (Figure 1b, Additional file 4). TMA-1 consisted of 113 patients while the second, TMA-2, consisted of 141 primary breast cancer tissue cores. TMA-1 consisted of both low grade (Grade 1, *n *= 15) and high grade (Grade 3, *n *= 74) ductal carcinomas [29]. Within this dataset, CrkI/II protein was significantly elevated in Grade 3 tumors versus Grade 1 tumors (Figure 1b). As expected, Ki67 antigen, a measure of proliferative index, also correlated strongly with Grade 3 tumors (Figure 1b). Importantly, within the high grade basal tumors in both TMA datasets, CrkI/II and CrkL protein levels correlated with proliferation, as assessed using Kendall's tau non-parametric statistical analysis (Table 1), demonstrating a strong link between elevated Crk protein and an aggressive tumor phenotype. Together, these observations identify a significant correlation of elevated Crk protein with high grade and poor outcome subtypes in human breast cancer.

**Table 1:** Crk protein expression correlates with Ki67 within the basal subtype

Antibody	Correlation to Ki67 proliferation index (% cells Ki67 positive)	
CrkI/II (TMA-1)	Correlation Coefficient	0.289
	*P*-value (2 tailed)	0.019
	number	43
CrkI/II (TMA-2)	Correlation Coefficient	0.323
	*P*-value (2 tailed)	0.016
	number	28
CrkII (TMA-2)	Correlation Coefficient	0.483
	*P*-value (2 tailed)	0.00024
	number	29
CrkL (C-20) (TMA-2)	Correlation Coefficient	0.483
	*P*-value (2 tailed)	0.00024
	number	29
CrkL (H-62) (TMA-2)	Correlation Coefficient	0.547
	*P*-value (2 tailed)	0.00003
	number	29

CrkI/II expression was examined in relation to Ki67 expression within 43 basal tumors using TMA-1. TMA-2 was utilized to examine Crk protein expression (using four different Crk antibodies recognizing CrkI/II, CrkII or CrkL) relative to Ki67 within 29 basal tumors. Kendall's tau non-parametric statistical analysis was used to assess for significance. TMA, tissue microarray.

### Crk proteins are required for cancer cell cytoskeletal organization, migration and invasion

**Figure 2:**
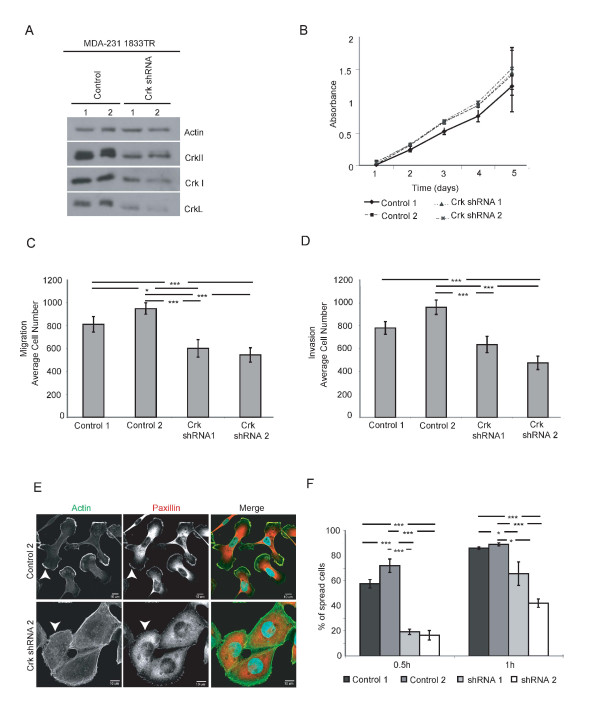
**Knockdown of Crk alters migration and invasion, cell morphology and adhesion**. Western blot of Crk proteins from whole cell lysates with actin as loading control (**A**). Alamar blue assay (*n *= 3) of control and Crk shRNA cells (**B**). Control and Crk shRNA cells were analyzed for migration (**C**) and invasion (**D**) using transwells (*n *= 3). Control and Crk shRNA cells plated on fibronectin coated coverslips (63x). Control cells exhibit lamellipodia (white arrowheads) compared to Crk shRNA cells, which lack these structures (white arrowheads). Cells co-stained with anti-paxillin and phalloidin. Scale bars represent 10 μm (**E**). Cell spreading (*n *= 3) was quantified by counting the total number of spread cells versus total cell number (**F**). Error bars represent SEM. Statistically significant data illustrated as (* = *P *< 0.05, ** = *P *< 0.01, *** = *P *< 0.001).

As elevated Crk proteins and a Crk gene signature are associated with an aggressive and proliferative basal breast cancer phenotype as well as basal breast cancer cell lines, we sought to examine the functional significance of Crk proteins in integrating signals for breast cancer progression. Thus, shRNAs were utilized to knock down all three Crk proteins in MDA-231 1833TR cells, a metastatic subpopulation of the basal MDA-231 cell line, that has a high affinity to form bone metastases [15]. Using CrkI/II and CrkL shRNA in pSuper and LMP vectors, respectively, multiple independent 1833TR cell lines were established with stable knockdown of Crk proteins (Figure 2a).

To first assess whether loss of Crk proteins affected proliferation, an Alamar Blue assay was performed. No significant differences in cell proliferation were observed (Figure 2b). In contrast, there was a significant decrease in cell migration and invasion towards 10% fetal bovine serum (FBS) in stable Crk knockdown cells compared to control cells (Figure 2c, d). Transient knockdown of CrkI/II and CrkL in combination resulted in a greater decrease in migration and invasion than single knockdown (CrkI/II or CrkL) alone (Additional file 5), supporting that Crk proteins have an additive role in promoting cell migration and invasion, and demonstrating a requirement to knock down all three Crk proteins within the 1833TR aggressive breast cancer cell line.

Since Crk proteins are implicated in integrating signals downstream of integrins, the importance of Crk for integrin engagement was assessed by plating cells on fibronectin. When cells were grown on fibronectin, staining of paxillin, a marker of focal adhesions, demonstrated that paxillin-positive adhesions were present within control and Crk knockdown cells (Figure 2e). However, whereas lamellipodia were identified in control cells, Crk knockdown cells failed to develop polarized membrane ruffles or lamellipodia and instead, dense cortical actin was observed (Figure 2e). In addition, delayed cellular spreading was found in Crk knockdown cells 0.5 and one hour post-plating (Figure 2f), establishing that diminished Crk expression decreased integrin-mediated spreading.

As Crk has been shown to regulate Rho GTPases involved in cell spreading and migration, we examined the activity of Rac1, which is known to regulate lamellipodia formation and cell migration [32]. Using a pull-down assay, small decreases in endogenous Rac GTP levels were consistently observed in Crk knockdown cells upon plating on collagen or upon stimulation with serum (Additional file 6). In contrast, a substantial decrease in Cdc42 activation was observed following serum stimulation (Additional file 6). Thus, knockdown of Crk proteins does not significantly disrupt Rac-dependent signals, but is required for activation of Cdc42 upon serum stimulation. In conclusion, alterations in cell morphology, diminished cellular spreading, and significant decreases in cell migration and invasion *in vitro *are caused by loss of Crk proteins and are associated with perturbations in Cdc42 activation.

### Crk proteins are required for breast cancer growth in the bone microenvironment

**Figure 3:**
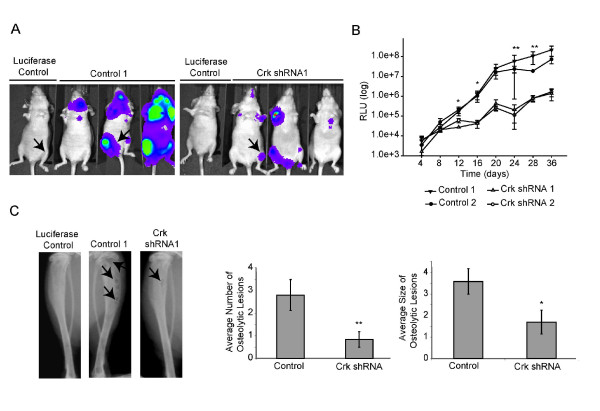
**Crk proteins are required for efficient breast cancer outgrowth in bone**. Outgrowth of 1833TR control (*n *= 7 for control 1, *n *= 4 for control 2) or Crk shRNA cells (*n *= 6 for Crk shRNA1, *n *= 6 for Crk shRNA 2) was measured by bioluminescence. Representative luciferase images at Day 40 are shown, closely resembling the mean bioluminescence values (**A**). Bioluminescence of positive limbs was quantified as relative luciferase units (RLU) over time. Averages from each cell line were plotted on a log scale +/- SEM (**B**). Representative X-ray images from negative control, control 1833TR and Crk shRNA mice. The average number and size of osteolytic lesions was blindly scored. Both control (*n *= 8) and Crk knockdown cohorts (*n *= 6) that exhibited osteolytic lesions were pooled for analysis (**C**). Statistically significant data illustrated as (* = *P *< 0.05, ** = *P *< 0.01, *** = *P *< 0.001).

Loss of Crk proteins impaired the ability of metastatic breast cancer cells to migrate, invade and adhere *in vitro*, thus, we examined how loss of Crk would affect bone metastasis *in vivo *following intra-cardiac injection, which takes into account several aspects of the metastatic cascade, including survival in the bloodstream, extravasation and secondary outgrowth. As stated previously, the MDA-231 1833TR cell line is a highly metastatic subpopulation when compared to the parental MDA-231 cell line and has a high affinity to form bone metastases [15]. Following intra-cardiac injection, cells are dispersed body-wide, and based on the observations that loss of Crk affects cell migration and invasion, we hypothesized that Crk proteins may modulate the ability of cells to extravasate and initiate early colonization steps. The 1833TR cells express luciferase, allowing for imaging of metastatic capacity [16]. Ablation of Crk significantly diminished the growth of metastatic bone lesions as assessed by bioluminescence, which correlated with decreases in the overall number and size of osteolytic lesions, as determined by X-ray analysis (Figure 3a-c). Interestingly, several of the animals injected with Crk knockdown cells failed to develop any metastatic lesions, relative to control cells in which 100% of the animals developed metastatic bone lesions (Table 2).

**Table 2:** Comparison of 'take rates' between the control 1833TR cells and the Crk shRNA cells from the various *in vivo *experiments

*In vivo *assay	Control 1	Control 2	Crk shRNA1	Crk shRNA 2	Controls (Total)	Crk shRNA (Total)
Cardiac	7/7	4/4	5/6	3/6	11/11 (100%)	8/12 (66.7%)
Tibial	8/16	7/16	10/16	4/16	15/32 (46.9%)	14/32 (43.8%)
Mammary Fat Pad	9/10	8/10	6/8	3/8	17/20 (85%)	9/16 (56.3%)

Since intra-cardiac injection encompasses multiple steps of the metastatic cascade, we wanted to establish whether Crk knockdown affects tumor outgrowth in the bone microenvironment. The tibial injection model [33] was chosen to focus our investigation on the importance of Crk proteins in mediating tumor cell interactions with the microenvironment, thereby excluding any possible effects that loss of Crk may have on tumor cell survival in circulation, endothelial adhesion, and extravasation. When compared to control cells, loss of Crk decreased tumor outgrowth in the tibia, as measured by bioluminescence imaging (Additional file 7), which was associated with a moderate decrease in the overall number and size of osteolytic lesions (Additional file 7). Thus, Crk proteins are required for outgrowth of 1833TR breast cancer cells in the bone microenvironment. Since differences observed between control and Crk knockdown 1833TR cells were more dramatic in the intra-cardiac model, loss of Crk may also contribute to bone metastasis.

### Crk proteins are required for mammary tumor growth

**Figure 4:**
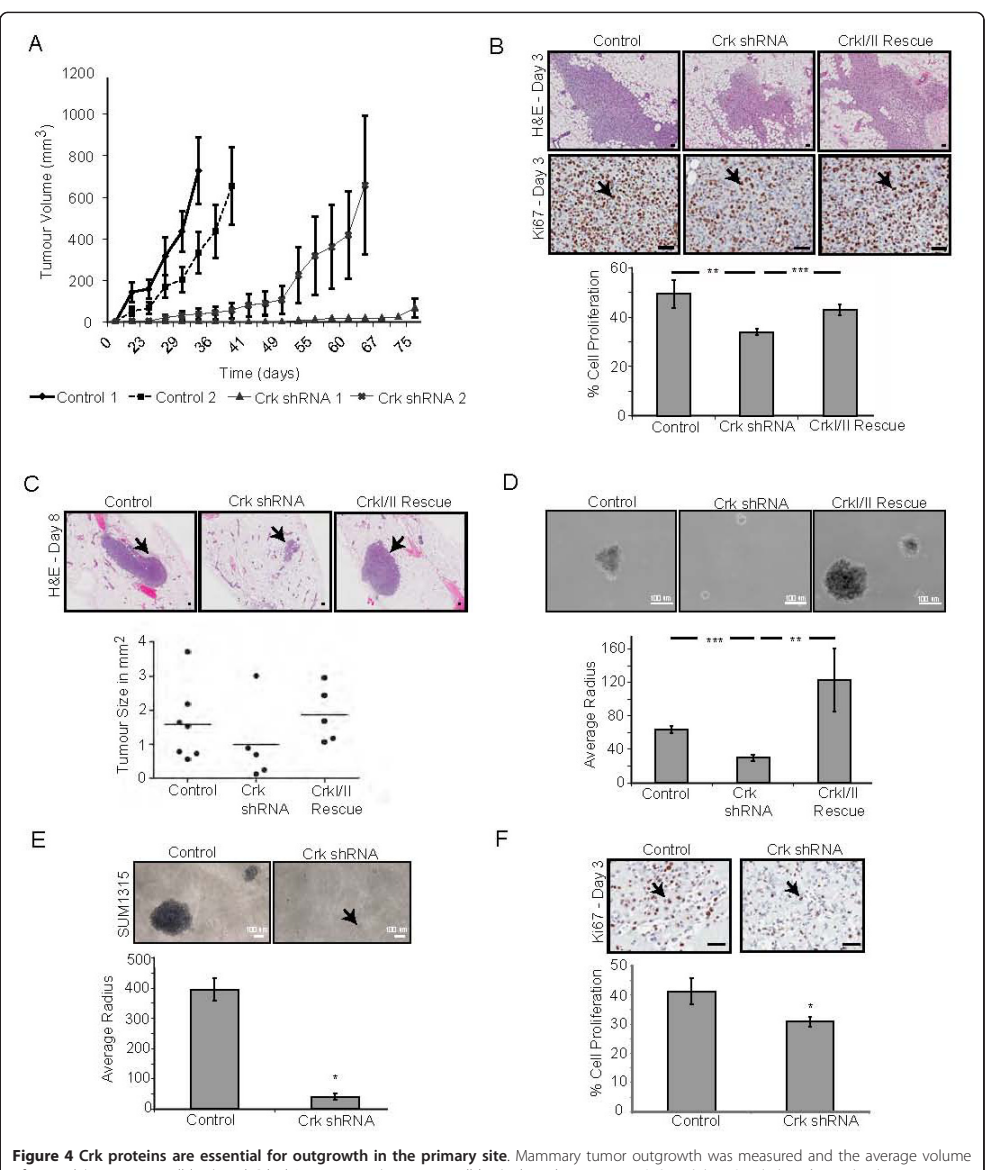
**Crk proteins are essential for outgrowth in the primary site**. Mammary tumor outgrowth was measured and the average volume of control (*n *= 10 per cell line) and Crk shRNA tumors (*n *= 8 per cell line) plotted over time +/- SEM (**A**). H & E (5x) and Ki67 (20x) staining were performed on sections from control, Crk shRNA and CrkI/II rescue samples three days post-injection. Scale bars 50 μm. Average cell proliferation +/- SEM was quantified using ImageScope (**B**). Representative H&E staining (2x) of lesion size eight days post-injection are shown. Scale bars 10 μm. Average lesion size eight days post-injection was plotted +/- SEM (**C**). Soft agar growth of control, Crk shRNA and CrkI/II rescue 1833TR cell lines; mean +/- SEM. Scale bars 100 μm (**D**). Soft agar growth of control and Crk shRNA SUM1315 cell lines; mean +/- SEM. Scale bars 100 μm. Soft agar quantified using Infinity Analyze (**E**). Ki67 (20x) staining of sections from control and Crk shRNA SUM1315 tumor samples three days post-injection. Scale bars 50 μm. Average cell proliferation +/- SEM quantified using ImageScope (**F**). Statistically significant data illustrated as (* = *P *< 0.05, ** = *P *< 0.01, *** = *P *< 0.001).

Since a significant delay in the outgrowth of 1833TR cells was observed in the bone microenvironment, we investigated whether loss of Crk would affect outgrowth in the primary site, the mammary fat pad. Similar to the intra-cardiac injection, seven out of the 16 animals injected with Crk knockdown cells failed to develop mammary tumors that reached a detectable size and the tumors that did derive were significantly delayed, taking twice as long as control cells to reach 500 mm^3 ^(Figure 4a, Table 2). Importantly, endpoint mammary tumors derived from control and Crk shRNA cells expressed similar levels of Crk protein (Additional file 8), indicating that Crk knockdown cells fail to form tumors and selective pressures exist within tumors to lose expression of Crk-targeting shRNAs, allowing re-expression of Crk proteins for tumor progression.

To investigate how mammary tumor outgrowth is altered by Crk knockdown, 1833TR cells were analyzed three days post-injection for proliferation and apoptosis. Relative to control cells, Crk knockdown significantly decreased proliferation, as assessed through Ki67 staining (Figure 4b) but not apoptosis (Additional file 8). Importantly, re-expression of non-shRNA targeted CrkI/II rescued *in vivo *proliferation, indicating a specific requirement of CrkI/II for sustained tumor growth (Figure 4b, Additional file 8). Analysis of microscopic lesions eight days post-injection revealed smaller lesions derived from Crk knockdown cells relative to control and CrkI/II rescue cells, highlighting the importance of Crk for initiation and maintenance of 1833TR induced tumors (Figure 4c). These data were unexpected considering Crk knockdown cells grow at similar rates to control cells *in vitro *(Figure 2b). Hence, we examined whether diminished Crk expression affects anchorage-independent growth. Loss of Crk significantly impaired the ability of 1833TR cells to grow in soft agar compared to control cells and re-expression of CrkI/II proteins rescued this phenotype (Figure 4d), consistent with a specific requirement for Crk in anchorage-independent growth.

To address whether the phenotypes observed upon Crk knockdown are specific to 1833TR cells, Crk knockdown was established in another basal breast cancer cell line, SUM1315. In a similar manner to 1833TR, Crk knockdown significantly attenuated anchorage-independent growth and perturbed *in vivo *proliferation of SUM1315 cells when injected into the mammary fat pad, confirming that Crk is required for tumor progression in other basal breast tumor cell lines (Figure 4e, f, Additional file 8). Loss of Crk also significantly impaired the ability of SUM1315 cells to migrate and invade (Additional file 8), meaning that Crk knockdown severely impairs the growth, migration, invasion, and proliferation of several basal breast tumor cells.

### Loss of Crk leads to decreased p130Cas phosphorylation

**Figure 5:**
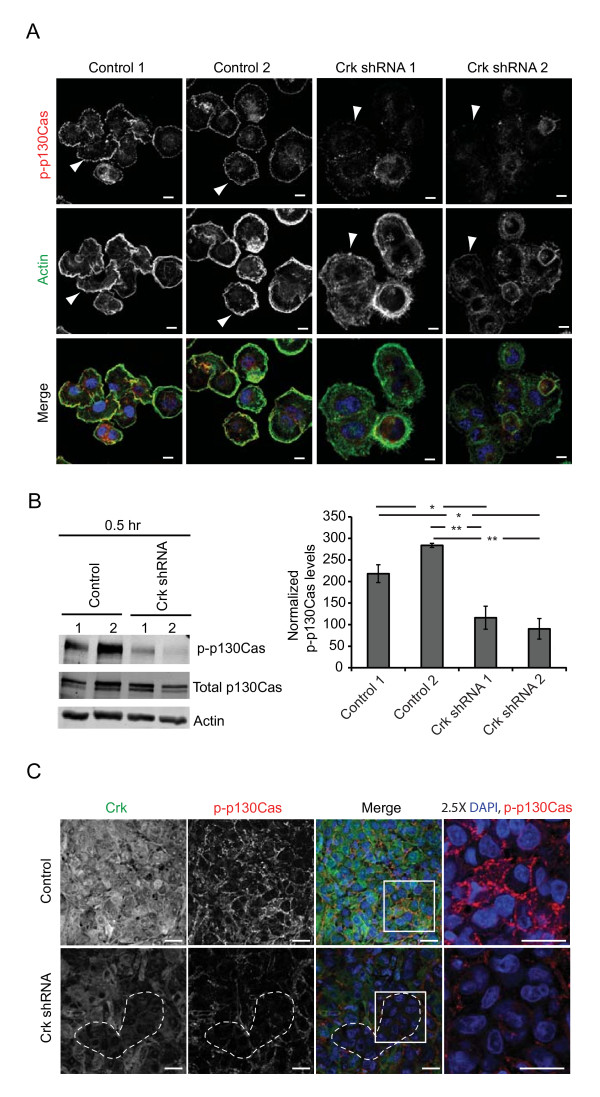
**Crk knockdown alters p130Cas phosphorylation**. Control and Crk shRNA MDA-231 1833TR cells plated on fibronectin one hour before fixation; stained for phospho-p130Cas and phalloidin. Phospho-p130Cas is prominent at membrane ruffles in control cells (white arrowheads), but cytosolic in Crk shRNA cells, with decreased accumulation of phalloidin at cell periphery (white arrowheads). Scale bars 10 μm (**A**). Western blot of proteins from control and Crk shRNA MDA-231 1833TR cells plated for 0.5 hour on fibronectin were quantified using Odyssey software. Phospho-p130Cas levels were normalized to total p130Cas levels (*n *= 3) (**B**). Immunofluorescence of CrkI/II and phosphorylated p130Cas was performed on sections from control and Crk shRNA MDA-231 1833TR samples three days post-injection. Scale bars 20 μm (**C**).

As previously mentioned, Crk adaptor proteins are known downstream integrators of integrin-mediated signaling and several studies have linked integrin signaling to tumor progression [34-36]. To understand the biochemical consequences of the diminished cell spreading and migration following Crk ablation, as well as the perturbations in tumor outgrowth, we examined known Crk-dependent signaling pathways associated with integrin activation. Integrin signaling induces phosphorylation of p130Cas (Crk-associated substrate), a scaffolding protein implicated in actin cytoskeleton reorganization, cell migration and spreading [37]. p130Cas was originally identified as a Crk SH2 domain-binding protein whose tyrosine phosphorylation is elevated upon v-Crk transformation of fibroblasts [37]. Immunofluorescence staining and confocal imaging of cells, one hour post-plating on fibronectin, revealed reduced phospho-p130Cas staining in Crk knockdown 1833TR cells relative to control cells. Interestingly, phospho-p130Cas was found predominantly in the cytoplasm rather than at membrane protrusions, as illustrated in control cells (Figure 5a). By Western blot analysis and quantification using digital software (Odyssey), phosphorylation of p130Cas was significantly reduced in Crk knockdown cells that demonstrated delayed cell spreading (Figure 5b). Thus, the delayed cell spreading correlated with decreased levels of phosphorylated p130Cas upon Crk knockdown and a diminished ability of phospho-p130Cas to engage in adhesions, supporting that Crk ablation impairs integrin signaling at least in part through reduced p130Cas phosphorylation and localization.

In order to assess whether impaired integrin signaling correlated with the decreases observed in tumor outgrowth, 1833TR and SUM1315 tumor lesions derived three days post-injection were immunostained for phospho-p130Cas and CrkI/II protein expression. Diminished levels of phospho-p130Cas were detected within Crk knockdown lesions relative to control lesions, supporting a perturbation in downstream integrin signaling *in vivo *as well as *in vitro *(Figure 5c, Additional file 9). A similar correlation was also observed in human breast cancer tumors. Breast tumors expressing low levels of CrkI/II, as visualized by confocal microscopy, exhibited diminished phosphorylation of p130Cas (Additional file 10). Conversely, breast tumors expressing high levels of CrkI/II demonstrated robust tyrosine phosphorylation of p130Cas (Additional file 10). Hence, in human breast cancers, high Crk protein expression is associated with tyrosine phosphorylation of p130Cas and supports our *in vitro *data illustrating that loss of Crk is associated with reduced p130Cas phosphorylation.

## Discussion

Although elevated Crk proteins are documented in several human cancers, Crk expression and signaling had not been well characterized in human breast cancer. Thus, it is of particular significance that a gene signature derived following CrkII over-expression correlates with the basal subtype in both human patients and breast cancer cell lines, and is associated with poor prognosis and decreased survival [31]. Using immunohistochemistry, we observed elevated CrkI/II and CrkL proteins in both high-grade tumors and proliferative breast cancers of the triple negative subtype, consistent with elevated Crk proteins being associated with aggressive breast cancer. The transcriptional Crk signature was also associated with high tumor grade and poor 20-year survival in multiple gene expression datasets from breast cancers. In support of this, CrkI/II are over-expressed in poorly differentiated and highly proliferative lung tumors [14], implicating Crk proteins in the progression from early to advanced states of disease.

The molecular mechanisms governing the induction and progression of basal breast cancer are poorly understood, and targeted therapies are lacking. Notably, the aggressive MDA-231 1833TR and SUM1315 cells used in this study are derived from the Basal B breast cancer subtype, which is associated with the Crk gene signature [27]. Using these basal breast cancer cell lines as a model, we demonstrate that Crk proteins are critical for growth in the mammary fat pad and bone. Interestingly, all mammary tumors that form with long latency from Crk knockdown MDA-231 1833TR cells expressed Crk proteins at levels that were similar to tumors derived from control cells, indicating that strong selective pressures exist within tumors for re-expression of Crk proteins for tumor progression.

Loss of Crk proteins abrogated the ability of aggressive MDA-231 1833TR breast cancer cells to grow in the bone after both intra-cardiac and tibial injections. Since loss of Crk diminished outgrowth in the tibia as well as the mammary fat pad, Crk proteins are likely required for tumor outgrowth regardless of the microenvironment. This is consistent with observations that loss of CrkI/II decreased the ability of ovarian cancer cells to grow within the peritoneal cavity and that ectopic expression of the microRNA miR-126, whose putative target is Crk, prevented outgrowth of gastric cancer cells in the lung and outgrowth of breast cancer cells in the lung, bone and mammary fat pad [13,38,39]. These data have important implications, as Crk proteins are elevated in many cancer types, including breast [6,11,12,14].

Since differences in bioluminescence observed between Crk knockdown and control cells following tibial injection were not as dramatic as those observed after intra-cardiac injection, the effect on metastasis formation may not be solely attributed to the effect on tumor outgrowth. The progression of cancer to metastasis is dependent, in part, on the deregulation of signaling pathways involved in cell migration and invasion. Downstream of integrins, Crk proteins can mediate migration through their association with the scaffold protein, p130Cas and the Rac exchange factor Dock180 [34]. Through these interactions, a role for Crk has been proposed in the regulation of cell migration, invasion, and phagocytosis, several of which have been demonstrated *in vivo *in *Caenorhabditis elegans *[3]. In the basal breast tumor cells examined here, loss of Crk reduces cell migration and invasion. However, although Crk has been well characterized upstream of Rac [34], we do not observe a strong correlation between Crk knockdown and overall Rac GTP levels, as assessed through pulldown assays. This suggests that Crk may be unlinked from Rac activation in the MDA-231 1833TR cell line or that Crk may affect the subcellular localization of Rac1 rather than its activation [40]. MDA-231 cells express mutant Ras (KRas G13D) [41], which is known to activate PI3K signaling, that can subsequently activate the Rac guanine nucleotide exchange factors, Sos and Vav1 [42] providing an alternative mechanism to activate Rac. However, these are insufficient to promote cell migration and invasion following Crk knockdown. A similar small reduction in Rac activation has been observed upon CrkI/II knockdown in MCAS ovarian cancer cells [38], further supporting the idea that Crk represents only one of several pathways that can activate Rac1.

Despite the small decreases in Rac activity, we observed a significant decrease in Cdc42 GTP levels in Crk knockdown cells upon serum stimulation. Cdc42 is involved in filopodia formation, as well as cell polarity and invadopodia [43] and has also been implicated in regulating Ras-mediated cell transformation [44]. Thus, our data support studies that identify Crk in signaling upstream of Cdc42 [45] and demonstrates that 1833TR breast tumor cells are dependent, at least in part, on Crk for activation of this GTPase, providing a potential mechanism for diminished *in vivo *tumor growth, as well as cell migration and invasion that are required for metastatic spread.

A role for integrin signaling in breast tumor progression is supported by studies demonstrating that β1 integrin inhibitory antibody decreased the number and size of mammary tumors and suppressed the growth of MDA-231 cells cultured in 3D extracellular matrix through decreased proliferation and enhanced apoptosis [35,36]. Additional studies examining metastases determined that impairment of integrin signaling is required, not for extravasation, but secondary outgrowth, highlighting the importance of integrin dependent signals for proliferation and survival *in vivo *[46,47]. Our studies identify a key role for Crk in this process. Following Crk knockdown, integrin-dependent cell spreading and tyrosine phosphorylation of p130Cas, a downstream target following integrin engagement, are impaired, and both MDA-231 and SUM1315 cells with Crk knockdown have decreased ability to grow in soft agar and impaired tumor outgrowth associated with diminished proliferation *in vivo*.

Crk proteins may be necessary to sustain the phosphorylation status of their SH2 domain-binding partners. Cells transformed with v-crk show elevated levels of tyrosine phosphorylation of Crk SH2 domain-binding proteins, including p130Cas [1,48-51] and CrkI/II is required for sustained phosphorylation of SH2 domain-binding protein Gab1 in response to growth factor stimulation [52]. In support of a requirement for Crk proteins for p130Cas signaling, we observe decreased tyrosine phosphorylation and mislocalization of p130Cas from the plasma membrane in basal breast cancer cells *in vitro*, as well as short term tumor assays *in vivo *following Crk knockdown. Decreased phosphorylation of p130Cas was also observed in human breast cancer tissue biopsies that displayed low levels of CrkI/II proteins, whereas elevated levels of p130Cas tyrosine phosphorylation correlated with high levels of Crk supporting the idea that Crk levels modulate signaling through p130Cas in human breast cancer *in vivo*.

## Conclusions

Elevated levels of Crk proteins (CrkI, CrkII, CrkL) are observed in human cancers, including those of epithelial origin, such as breast. Despite *in vitro *studies on cell transformation, migration and invasion, the precise role of Crk adaptor proteins in epithelial derived breast cancers and their correlation to human clinical parameters *in vivo *is poorly understood. We demonstrate the clinical significance of Crk proteins in human breast cancer using TMAs (*n *= 254), revealing a significant association between Crk protein expression with highly proliferative tumors and basal breast cancers of poor outcome. Moreover, a gene expression signature derived following CrkII over-expression significantly correlated with basal breast cancer and poor outcome. As a model to study the functional significance of Crk adaptor proteins within basal breast cancer, we utilized two basal breast cancer cell lines and demonstrated that Crk proteins are important for cellular adhesion, migration, invasion and *in vivo *proliferative breast tumor outgrowth. The data generated from this study identify Crk proteins, or Crk-dependent signaling pathways, such as p130Cas, as potential targets for the basal subtype of breast cancer.

## Abbreviations

Cas: Crk-associated substrate; Cdc42: cell division cycle 42; Crk: CT10 regulator of kinase; Crk-L: Crk-Like; Dock180: dedicator of cytokinesis 1; ER: estrogen receptor; FBS: fetal bovine serum; GTP: guanosine triphosphate; H & E: hematoxylin and eosin; HER2: human epidermal growth factor receptor 2; LMP: MSCV-LTR-mir30-PIG; mRNA: messenger ribonucleic acid; MUHC: McGill University Health Centre; PI3K: phosphoinositide 3-kinase; PR: progesterone receptor; Rac1: ras-related C3 botulinum toxin substrate 1; RLU: relative luciferase units; RNAi: RNA interference; SDS-PAGE: sodium dodecyl sulfate polyacrylamide gel electrophoresis; SEM: standard error of the mean; SH2: Src homology 2; SH3: Src homology 3; shRNA: short hairpin RNA; TMA: tissue microarray; TN: triple negative; TR: triple reporter.

## Competing interests

The authors declare that they have no competing interests.

## Authors' contributions

KEF designed and performed the research, analyzed data and wrote the paper. ESB designed and performed experiments and analyzed data. CVR, AM, HZ, DZ, AM and AAG performed experiments. JM, AMM, LG, SC and MH analyzed data. PS contributed reagents and analyzed data. MP analyzed data and wrote the paper. All authors contributed to the editing of the manuscript.

## Supplementary Material

Additional file 1 **The Crk gene signature is composed of 151 genes that are differentially expressed following CrkII over-expression in T47D cells**. Click here for file

Additional file 2 **Additional microarray datasets illustrating an association of the Crk gene signature with basal molecular subtype and high tumor grade**. Heatmaps of the Crk signature in the Ivshina, Loi, Parker and Wang breast cancer datasets. The Crk signature is derived from CrkII over-expressing T47D cell lines. The bars on the left indicate if the gene had increased or decreased expression in the CrkII cell line compared to the control. The patients are ordered by correlation of the expression of these 151 genes in the patients sample to the Crk signature. Blue indicates increased expression whereas yellow indicates decreased expression. The association between patient ordering, molecular subtype and grade was determined using a Krusal-Wallis rank sum test. Click here for file

Additional file 3 **There is an association with the Crk gene signature and the basal molecular subtypes found within breast cancer cell lines**. Heatmap of the Crk signature in the Neve breast cancer cell line dataset. The Crk signature is derived from CrkII over-expressing T47D cell lines. The bars on the left indicate if the gene had increased or decreased expression in the CrkII cell line compared to the control T47D cell line. The breast cancer cell lines are ordered by correlation of the expression of these 151 genes in the cell line to the Crk signature. Blue indicates increased expression whereas yellow indicates decreased expression. Within the gene cluster category, blue represents luminal, pink represents Basal A and dark red represents Basal B subtypes. Click here for file

Additional file 4 **CrkI/II positivity within human breast cancer tissue microarrays**. A representative image of CrkI/II immunohistochemical staining within TMA#1. Image taken using ScanScope software. Click here for file

Additional file 5 **All 3 Crk proteins are required for sufficient decreases in cell migration and invasion**. MDA-231 1833TR cells were transiently transfected and analyzed for their migration (A) and invasion (B) capacity in the presence of either mock transfected, scramble siRNA, CrkI/II, CrkL siRNA or both CrkI/II and CrkL siRNA in combination. Image analysis of these assays was carried out using Scion Image software. A minimum of three experiments were performed. Error bars represent the standard error of the three experiments (A, B). Western blot analysis of proteins from whole cell lysates (MDA-231 1833TR) with an anti-CrkI/II or anti-CrkL sera was performed and actin protein levels were used as a loading control (C). All data that are statistically significant is illustrated as follows (* represents *P *< 0.05), ** represents *P *< 0.01, *** represents *P *< 0.001). Click here for file

Additional file 6 **Crk knockdown alters Cdc42 activation but does not significantly impact Rac activation**. Rac1 pulldown experiments were performed after a four hour serum starvation, followed by lysis 30 minutes post-plating on collagen. All samples were analyzed at the same time, allowing for comparisons. The GTP bound form of endogenous Rac was precipitated by GST-CRIB and probed for anti-Rac1 sera. Total endogenous levels of Rac were visualized by immunoblot. Activated levels of Rac1 were quantified and compared to total Rac1 levels (*n *= 6) (A). In response to serum, Rac and Cdc42 activation was assessed utilizing GST-Pak-PBD or GST-WASP as binding partners for GTP loaded Rac and Cdc42 respectively. Pulldown experiments were performed after a four hour serum starvation, followed by 15 minutes serum stimulation. All samples were analyzed at the same time, allowing for comparisons. The GTP bound form of endogenous Rac was precipitated by GST-Pak-PBD and probed for anti-Rac1 sera. Total endogenous levels of Rac were visualized by immunoblot. Activated levels of Rac1 were quantified and compared to total Rac1 levels (*n *= 4) (B). The GTP bound form of endogenous Cdc42 was precipitated by GST-WASP and probed for anti-Cdc42 sera. Total levels of endogenous Cdc42 were visualized by immunoblot. Activated levels of Cdc42 were quantified as described above (C). Error bars represent SEM. All data that are statistically significant is illustrated as follows (* represents *P *< 0.05), ** represents *P *< 0.01, *** represents *P *< 0.001). Click here for file

Additional file 7 **Crk proteins are required for efficient outgrowth of breast cancer cells in the tibia**. MDA-231 1833TR control cells or those expressing Crk shRNA were injected directly into the tibia and tumor outgrowth was measured by bioluminescence imaging. Tumor outgrowth within the tibia was quantified using limbs positive for bioluminescent activity, measured as relative luciferase units (RLU) over time and plotted on a log scale as the mean +/- SEM (A). The average number and size of osteolytic lesions from pooled control (*n *= 9) and Crk knockdown samples (*n *= 5) was quantified and plotted with SEM (B). All data that is statistically significant is illustrated as follows (* represents *P *< 0.05), ** represents *P *< 0.01, *** represents *P *< 0.001). Click here for file

Additional file 8 **Crk expression and growth kinetics *in vitro *and *in vivo***. Western blot analysis of Crk protein expression levels from tumor endpoint (A). Paraffin embedded sections of mammary tumors derived from 1833TR control and Crk shRNA cells were stained for Ki67 (positive staining represented by black arrows) and TUNEL (positive staining represented by black arrows). No significant differences were observed between control and Crk knockdown tumors for Ki67 and TUNEL. All images taken at 20x where the scale bar represents 50 μm. Arrows represent area of insert (B). CrkI/II rescue was examined via Western blot analysis of whole cell lysates (MDA-231 1833TR) with an anti-CrkI/II or anti-CrkL sera. Actin protein levels were used as a loading control (C). Paraffin embedded sections of mammary tumors derived from 1833TR control, Crk shRNA and CrkI/II rescue cells were stained for apoptosis via TUNEL staining and quantified using ImageScope (D). Crk knockdown of SUM1315 cells was examined via Western blot analysis of whole cell lysates with an anti-CrkI/II or anti-CrkL sera. Alpha-tubulin protein levels were used as a loading control (E). Control and Crk shRNA SUM1315 cells (8 × 10^4 ^cells) were plated on transwells and were analyzed for migration (F) and invasion (G) towards 10% FBS 24 hours post-plating then quantified using ImageScope software (*n *= 3). All data that is statistically significant is illustrated as follows (* represents *P *< 0.05), ** represents *P *< 0.01, *** represents *P *< 0.001). Click here for file

Additional file 9 **Phosphorylated p130Cas is diminished in lesions derived from Crk knockdown cell lines**. Immunofluorescence of CrkI/II and phosphorylated p130Cas was performed on paraffin embedded sections from control and Crk shRNA MDA-231 1833TR samples three days post-injection. Scale bars 20 μm (A). Immunofluorescence of CrkI/II and phosphorylated p130Cas was performed on paraffin embedded sections from control and Crk shRNA SUM1315 samples 3 days post-injection. Scale bars 20 μm (A). Click here for file

Additional file 10 **Phosphorylated p130Cas expression correlates with CrkI/II expression in human breast cancer tumors**. Immunofluoresence of CrkI/II and phosphorylated p130Cas was performed on frozen human basal breast cancer tissue. CrkI/II mRNA expression from these patients was also assessed by microarray analysis. TN = triple negative, ER = estrogen receptor. Scale bars 20 μm (A). Click here for file
