# Supplementary material for: Crk adaptor proteins act as key signaling integrators for breast tumorigenesis
Source: Breast Cancer Res. 2012 May 8;14(3):R74. doi: 10.1186/bcr3183 (PMC3446336; doi:10.1186/bcr3183)
Supplement: Additional file 1 — The Crk gene signature is composed of 151 genes that are differentially expressed following CrkII over-expression in T47D cells. [file bcr3183-S1.DOC]

**Additional File 1 –** The Crk gene signature is composed of 151 genes that are differentially expressed following CrkII over-expression in T47D cells.

| **AgilentProbeID** | **Agilent**  **GeneName** | **Description** | **logFC** |
| --- | --- | --- | --- |
| A_24_P187218 | PCDH9 | protocadherin 9 | 3.24 |
| A_23_P94501 | ANXA1 | annexin A1 | 2.51 |
| A_23_P170233 | CSTA | cystatin A (stefin A) | 2.42 |
| A_23_P114903 | HSPA6 | heat shock 70kDa protein 6 (HSP70B') | 2.29 |
| A_23_P169437 | LCN2 | lipocalin 2 | 2.23 |
| A_23_P19663 | CTGF | connective tissue growth factor | 2.17 |
| A_23_P204630 | NTN4 | netrin 4 | 2.09 |
| A_23_P125233 | CNN1 | calponin 1; basic; smooth muscle | 2.07 |
| A_23_P81219 | PLAC8 | placenta-specific 8 | 1.94 |
| A_23_P371729 | GJA5 | gap junction protein; alpha 5; 40kDa | 1.94 |
| A_23_P24129 | DKK1 | dickkopf homolog 1 (Xenopus laevis) | 1.91 |
| A_23_P62099 | MAGEC2 | melanoma antigen family C; 2 | 1.85 |
| A_24_P187799 | FLJ21986 | chromosome 7 open reading frame 58 | 1.82 |
| A_23_P121716 | ANXA3 | annexin A3 | 1.8 |
| A_24_P207995 | L1CAM | L1 cell adhesion molecule | 1.8 |
| A_32_P130734 | THC2724145 | NA | 1.76 |
| A_23_P107351 | NLRP1 | NLR family; pyrin domain containing 1 | 1.65 |
| A_23_P15101 | TMC5 | transmembrane channel-like 5 | 1.65 |
| A_23_P39955 | ACTG2 | actin; gamma 2; smooth muscle; enteric | 1.65 |
| A_32_P154473 | KIF5C | kinesin family member 5C | 1.63 |
| A_23_P161352 | PTPLA | protein tyrosine phosphatase-like (proline instead of catalytic arginine); member A | 1.62 |
| A_32_P107372 | GBP1 | guanylate binding protein 1; interferon-inducible; 67kDa | 1.62 |
| A_23_P42575 | CALD1 | caldesmon 1 | 1.58 |
| A_23_P16523 | GDF15 | growth differentiation factor 15 | 1.56 |
| A_23_P79302 | LOC130576 | hypothetical protein LOC130576 | 1.52 |
| A_23_P128974 | BATF | basic leucine zipper transcription factor; ATF-like | 1.43 |
| A_23_P126869 | PADI3 | peptidyl arginine deiminase; type III | 1.42 |
| A_24_P750817 | LOC147710 | hypothetical LOC147710 | 1.42 |
| A_24_P141707 | INHBE | inhibin; beta E | 1.42 |
| A_32_P68504 | KIAA1571 | zinc finger; DBF-type containing 2 | 1.4 |
| A_23_P252981 | ACE2 | angiotensin I converting enzyme (peptidyl-dipeptidase A) 2 | 1.39 |
| A_23_P89431 | CCL2 | chemokine (C-C motif) ligand 2 | 1.39 |
| A_23_P413641 | PREX1 | phosphatidylinositol 3;4;5-trisphosphate-dependent RAC exchanger 1 | 1.37 |
| A_24_P887857 | LOC650517 | keratin 17 pseudogene 3 | 1.37 |
| A_23_P35617 | PLCE1 | phospholipase C; epsilon 1 | 1.35 |
| A_23_P101564 | FGF21 | fibroblast growth factor 21 | 1.33 |
| A_23_P2674 | KRT4 | keratin 4 | 1.32 |
| A_23_P393620 | TFPI2 | tissue factor pathway inhibitor 2 | 1.29 |
| A_23_P27556 | EMR1 | egf-like module containing; mucin-like; hormone receptor-like 1 | 1.3 |
| A_23_P134734 | FLJ20366 | Golgi-localized protein | 1.28 |
| A_24_P222872 | UGT1A6 | UDP glucuronosyltransferase 1 family; polypeptide A6 | 1.26 |
| A_23_P207905 | SECTM1 | secreted and transmembrane 1 | 1.25 |
| A_23_P52207 | BAMBI | BMP and activin membrane-bound inhibitor homolog (Xenopus laevis) | 1.25 |
| A_23_P365738 | ARC | activity-regulated cytoskeleton-associated protein | 1.25 |
| A_23_P168836 | PTK2B | PTK2B protein tyrosine kinase 2 beta | 1.24 |
| A_23_P127948 | ADM | adrenomedullin | 1.23 |
| A_23_P163697 | SYT17 | synaptotagmin XVII | 1.21 |
| A_23_P56898 | KYNU | kynureninase (L-kynurenine hydrolase) | 1.2 |
| A_23_P148609 | PLAC1 | placenta-specific 1 | 1.2 |
| A_23_P80491 | RBP2 | retinol binding protein 2; cellular | 1.17 |
| A_24_P226008 | MGLL | monoglyceride lipase | 1.16 |
| A_24_P11384 | ERRFI1 | ERBB receptor feedback inhibitor 1 | 1.16 |
| A_23_P18078 | RARRES1 | retinoic acid receptor responder (tazarotene induced) 1 | 1.16 |
| A_24_P233786 | FAM129A | family with sequence similarity 129; member A | 1.14 |
| A_24_P196528 | CRB1 | crumbs homolog 1 (Drosophila) | 1.13 |
| A_23_P353524 | IVL | involucrin | 1.13 |
| A_23_P96158 | KRT17 | keratin 17 | 1.12 |
| A_32_P135336 | LOC388242 | LOC112869 pseudogene | 1.13 |
| A_23_P162579 | HSPB8 | heat shock 22kDa protein 8 | 1.13 |
| A_23_P104996 | BEST1 | bestrophin 1 | 1.12 |
| A_23_P136041 | PRKG1 | protein kinase; cGMP-dependent; type I | 1.11 |
| A_23_P148568 | NXF2 | nuclear RNA export factor 2 | 1.11 |
| A_23_P58676 | C5orf23 | chromosome 5 open reading frame 23 | 1.1 |
| A_23_P327380 | TP73L | tumor protein p63 | 1.11 |
| A_24_P11825 | CCR2 | chemokine (C-C motif) receptor 2 | 1.11 |
| A_24_P370946 | CYR61 | cysteine-rich; angiogenic inducer; 61 | 1.1 |
| A_24_P355944 | EFNB2 | ephrin-B2 | 1.1 |
| A_23_P159325 | ANGPTL4 | angiopoietin-like 4 | 1.09 |
| A_23_P111240 | PHACTR2 | phosphatase and actin regulator 2 | 1.09 |
| A_32_P42684 | SLC7A11 | solute carrier family 7; (cationic amino acid transporter; y+ system) member 11 | 1.08 |
| A_24_P77082 | KMO | kynurenine 3-monooxygenase (kynurenine 3-hydroxylase) | 1.08 |
| A_24_P206776 | CRYAB | crystallin; alpha B | 1.09 |
| A_23_P452 | DDR2 | discoidin domain receptor tyrosine kinase 2 | 1.08 |
| A_23_P360379 | EGLN3 | egl nine homolog 3 (C. elegans) | 1.07 |
| A_23_P215111 | ATP6V0A4 | ATPase; H+ transporting; lysosomal V0 subunit a4 | 1.06 |
| A_24_P33446 | LOC338651 | keratin associated protein 5-2 | 1.06 |
| A_24_P940166 | PAPSS2 | 3'-phosphoadenosine 5'-phosphosulfate synthase 2 | 1.06 |
| A_23_P87941 | ALDH1L2 | aldehyde dehydrogenase 1 family; member L2 | 1.05 |
| A_23_P203267 | TRIM29 | tripartite motif-containing 29 | 1.04 |
| A_23_P501985 | CSF2RA | colony stimulating factor 2 receptor; alpha; low-affinity (granulocyte-macrophage) | 1.03 |
| A_23_P29096 | PDE9A | phosphodiesterase 9A | 1.04 |
| A_23_P435394 | PTGER4 | prostaglandin E receptor 4 (subtype EP4) | 1.03 |
| A_23_P321892 | HRG | histidine-rich glycoprotein | 1.03 |
| A_23_P125705 | NAP1L2 | nucleosome assembly protein 1-like 2 | 1.03 |
| A_23_P335239 | GAB1 | GRB2-associated binding protein 1 | 1.03 |
| A_23_P121011 | AXUD1 | AXIN1 up-regulated 1 | 1.03 |
| A_23_P327451 | NPR3 | natriuretic peptide receptor C/guanylate cyclase C (atrionatriuretic peptide receptor C) | 1.03 |
| A_23_P82503 | PEG10 | paternally expressed 10 | 1.03 |
| A_23_P434809 | S100A8 | S100 calcium binding protein A8 | 1.02 |
| A_23_P134113 | C6orf192 | chromosome 6 open reading frame 192 | 1.02 |
| A_23_P111701 | GNG11 | guanine nucleotide binding protein (G protein); gamma 11 | 1.01 |
| A_23_P254831 | MAGEB2 | melanoma antigen family B; 2 | 1.01 |
| A_23_P154605 | SULF2 | sulfatase 2 | -2.83 |
| A_23_P216023 | ANGPT1 | angiopoietin 1 | -2.45 |
| A_23_P144929 | RANBP3L | RAN binding protein 3-like | -2.33 |
| A_24_P197537 | PDE8B | phosphodiesterase 8B | -2.31 |
| A_23_P41344 | EREG | epiregulin | -2.19 |
| A_24_P55295 | GJA1 | gap junction protein; alpha 1; 43kDa | -2.04 |
| A_23_P144980 | PIK3R1 | phosphoinositide-3-kinase; regulatory subunit 1 (alpha) | -2.02 |
| A_23_P105212 | THRSP | thyroid hormone responsive (SPOT14 homolog; rat) | -1.93 |
| A_23_P166929 | SERPINI1 | serpin peptidase inhibitor; clade I (neuroserpin); member 1 | -1.92 |
| A_23_P107911 | KLK10 | kallikrein-related peptidase 10 | -1.85 |
| A_24_P49267 | LOC646282 | similar to ZN-alpha-2-glycoprotein | -1.79 |
| A_32_P49199 | AL137566 | progesterone receptor | -1.79 |
| A_24_P215765 | ATP10A | ATPase; class V; type 10A | -1.79 |
| A_23_P144656 | CDH10 | cadherin 10; type 2 (T2-cadherin) | -1.75 |
| A_24_P412156 | CXCL12 | chemokine (C-X-C motif) ligand 12 (stromal cell-derived factor 1) | -1.72 |
| A_23_P363316 | HOXB5 | homeobox B5 | -1.7 |
| A_23_P329768 | GREB1 | GREB1 protein | -1.67 |
| A_23_P32577 | DACH1 | dachshund homolog 1 (Drosophila) | -1.62 |
| A_23_P105144 | SCUBE2 | signal peptide; CUB domain; EGF-like 2 | -1.6 |
| A_23_P48740 | DIO2 | deiodinase; iodothyronine; type II | -1.53 |
| A_23_P116249 | GRIK4 | glutamate receptor; ionotropic; kainate 4 | -1.48 |
| A_23_P326760 | MYRIP | myosin VIIA and Rab interacting protein | -1.46 |
| A_23_P156425 | MAN1A1 | mannosidase; alpha; class 1A; member 1 | -1.46 |
| A_23_P66682 | HOXB6 | homeobox B6 | -1.43 |
| A_23_P71270 | AZGP1 | alpha-2-glycoprotein 1; zinc-binding | -1.42 |
| A_24_P320124 | NPY5R | neuropeptide Y receptor Y5 | -1.42 |
| A_23_P30217 | SCGB3A1 | secretoglobin; family 3A; member 1 | -1.4 |
| A_24_P586712 | FAM79B | tumor protein p63 regulated 1 | -1.35 |
| A_24_P335781 | ADCYAP1 | adenylate cyclase activating polypeptide 1 (pituitary) | -1.31 |
| A_23_P19673 | SGK | serum/glucocorticoid regulated kinase 1 | -1.32 |
| A_23_P207939 | C18orf1 | chromosome 18 open reading frame 1 | -1.3 |
| A_23_P69699 | NPY1R | neuropeptide Y receptor Y1 | -1.28 |
| A_23_P167812 | RBM24 | RNA binding motif protein 24 | -1.27 |
| A_23_P144778 | CKMT2 | creatine kinase; mitochondrial 2 (sarcomeric) | -1.27 |
| A_23_P259071 | AREG | amphiregulin (schwannoma-derived growth factor) | -1.24 |
| A_23_P311818 | GHRH | growth hormone releasing hormone | -1.24 |
| A_24_P213478 | SEMA6A | sema domain; transmembrane domain (TM); and cytoplasmic domain; (semaphorin) 6A | -1.21 |
| A_23_P358714 | KIAA1324 | KIAA1324 | -1.17 |
| A_23_P31073 | MYB | v-myb myeloblastosis viral oncogene homolog (avian) | -1.17 |
| A_24_P212811 | ANKRD34 | ankyrin repeat domain 34A | -1.14 |
| A_24_P328657 | PEX5L | peroxisomal biogenesis factor 5-like | -1.13 |
| A_23_P159316 | BFSP2 | beaded filament structural protein 2; phakinin | -1.13 |
| A_23_P420863 | NOD2 | nucleotide-binding oligomerization domain containing 2 | -1.12 |
| A_32_P182299 | C1orf168 | chromosome 1 open reading frame 168 | -1.12 |
| A_24_P270496 | NOVA1 | neuro-oncological ventral antigen 1 | -1.11 |
| A_23_P500741 | CBFA2T3 | core-binding factor; runt domain; alpha subunit 2; translocated to; 3 | -1.11 |
| A_23_P110403 | PDLIM3 | PDZ and LIM domain 3 | -1.1 |
| A_23_P168351 | HEY2 | hairy/enhancer-of-split related with YRPW motif 2 | -1.1 |
| A_23_P11685 | PLA2G4A | phospholipase A2; group IVA (cytosolic; calcium-dependent) | -1.09 |
| A_23_P42811 | BCMP11 | anterior gradient homolog 3 (Xenopus laevis) | -1.09 |
| A_24_P152188 | PRICKLE2 | prickle homolog 2 (Drosophila) | -1.08 |
| A_23_P311912 | C14orf78 | AHNAK nucleoprotein 2 | -1.08 |
| A_23_P257763 | BOC | Boc homolog (mouse) | -1.07 |
| A_23_P427014 | CLDN8 | claudin 8 | -1.07 |
| A_23_P88559 | LIPC | lipase; hepatic | -1.04 |
| A_23_P2573 | TMEM117 | transmembrane protein 117 | -1.03 |
| A_24_P934387 | AL049270 | solute carrier family 5 (sodium/glucose cotransporter); member 12 | -1.02 |
| A_23_P144959 | CSPG2 | versican | -1.01 |
| A_23_P304450 | GATA6 | GATA binding protein 6 | -1.01 |
