# Supplementary figures and images for: Crk adaptor proteins act as key signaling integrators for breast tumorigenesis
Source: Breast Cancer Res. 2012 May 8;14(3):R74. doi: 10.1186/bcr3183 (PMC3446336; doi:10.1186/bcr3183)

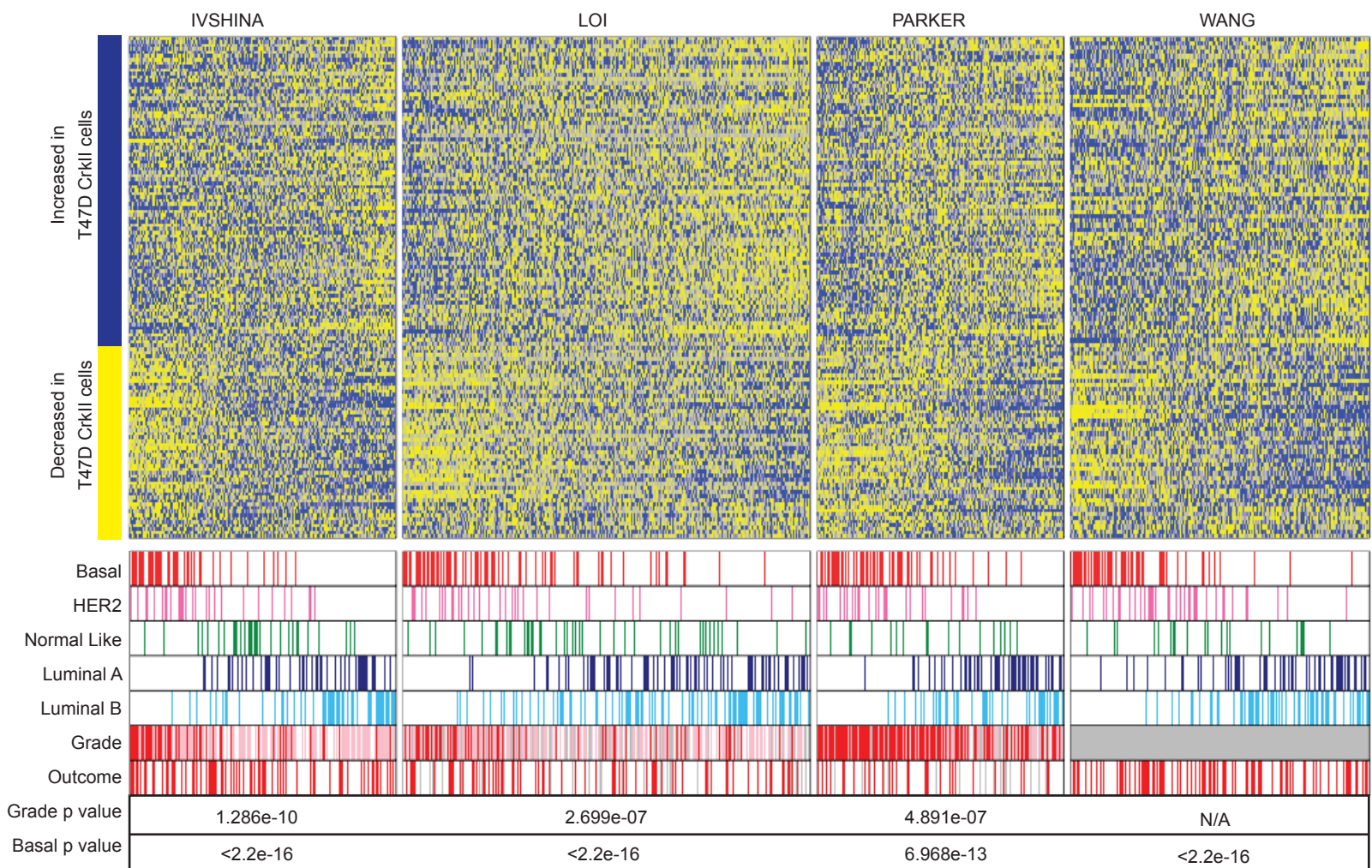

Supplement: Additional file 2 — Additional microarray datasets illustrating an association of the Crk gene signature with basal molecular subtype and high tumor grade. Heatmaps of the Crk signature in the Ivshina, Loi, Parker and Wang breast cancer datasets. The Crk signature is derived from CrkII over-expressing T47D cell lines. The bars on the left indicate if the gene had increased or decreased expression in the CrkII cell line compared to the control. The patients are ordered by correlation of the expression of these 151 genes in the patients sample to the Crk signature. Blue indicates increased expression whereas yellow indicates decreased expression. The association between patient ordering, molecular subtype and grade was determined using a Krusal-Wallis rank sum test. [file bcr3183-S2.PDF]

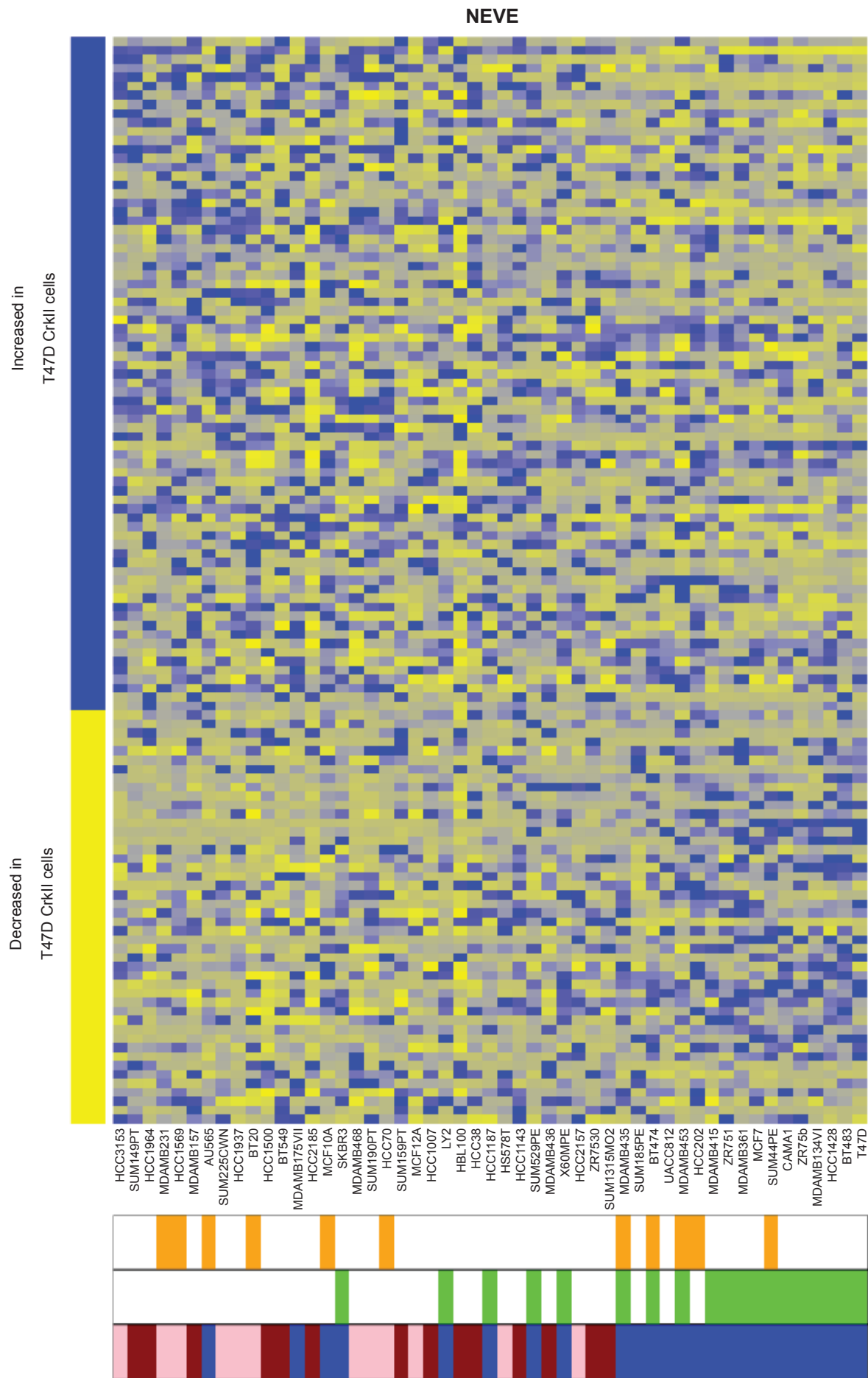

Supplement: Additional file 3 — There is an association with the Crk gene signature and the basal molecular subtypes found within breast cancer cell lines. Heatmap of the Crk signature in the Neve breast cancer cell line dataset. The Crk signature is derived from CrkII over-expressing T47D cell lines. The bars on the left indicate if the gene had increased or decreased expression in the CrkII cell line compared to the control T47D cell line. The breast cancer cell lines are ordered by correlation of the expression of these 151 genes in the cell line to the Crk signature. Blue indicates increased expression whereas yellow indicates decreased expression. Within the gene cluster category, blue represents luminal, pink represents Basal A and dark red represents Basal B subtypes. [file bcr3183-S3.PDF]

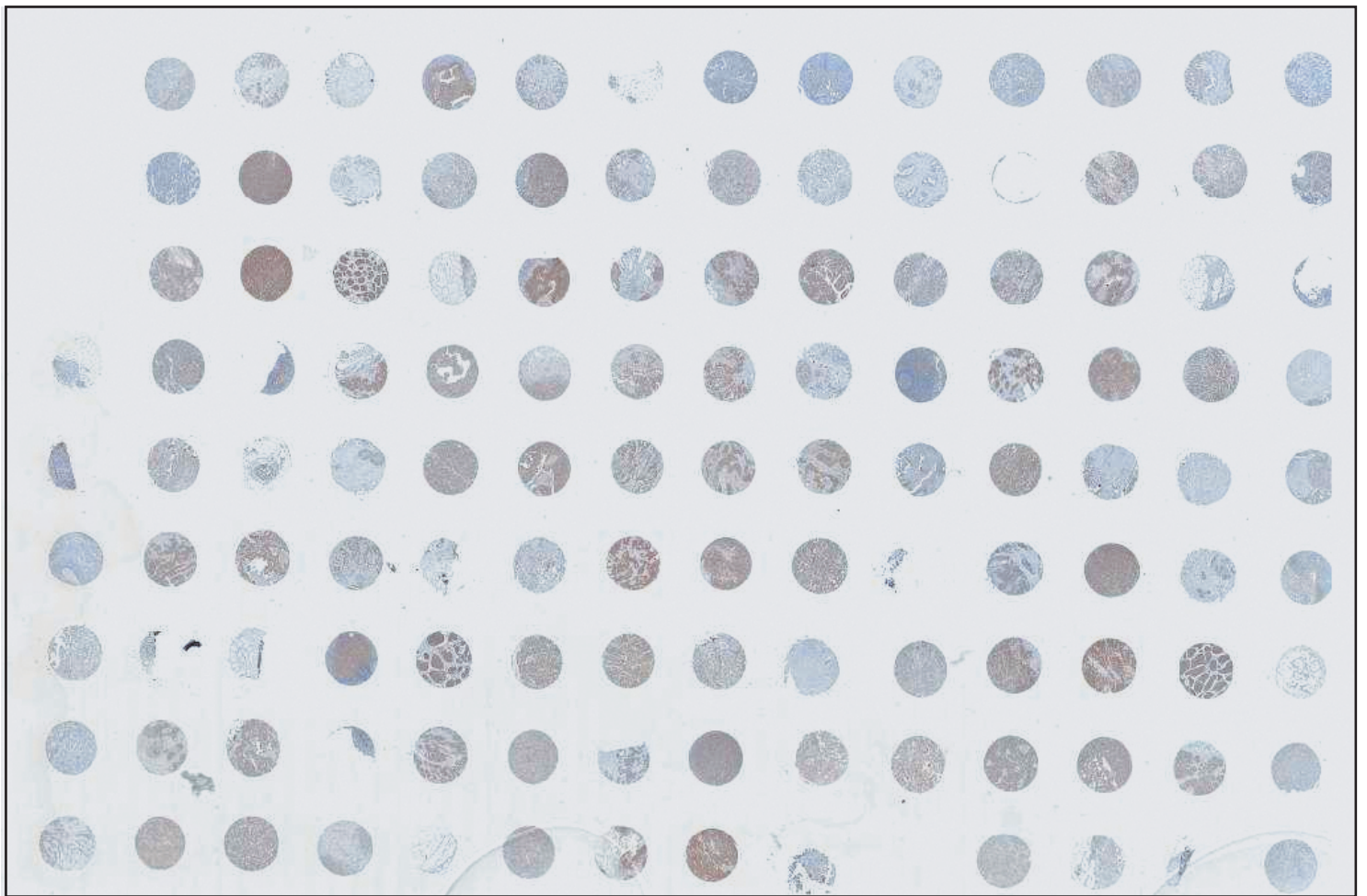

Supplement: Additional file 4 — CrkI/II positivity within human breast cancer tissue microarrays. A representative image of CrkI/II immunohistochemical staining within TMA#1. Image taken using ScanScope software. [file bcr3183-S4.PDF]

A

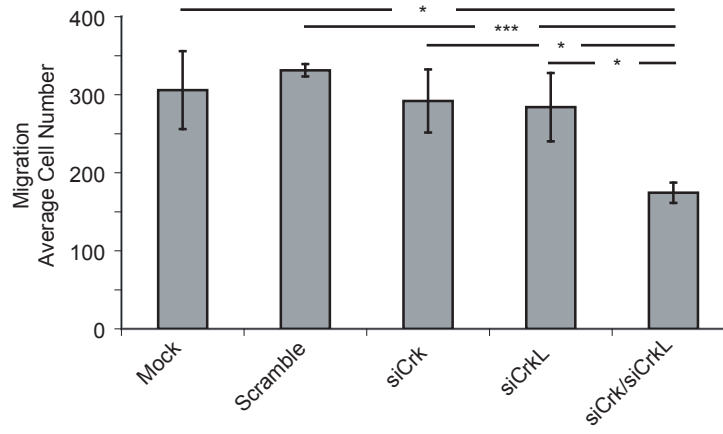

B

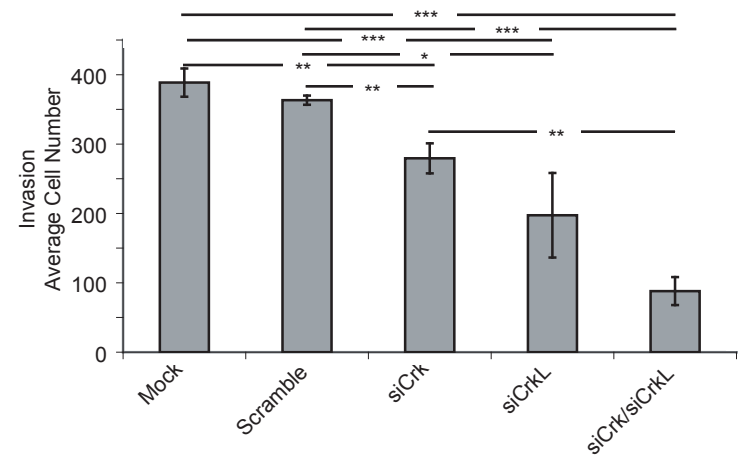

C

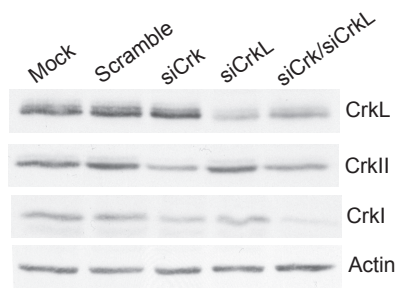

Supplement: Additional file 5 — All 3 Crk proteins are required for sufficient decreases in cell migration and invasion. MDA-231 1833TR cells were transiently transfected and analyzed for their migration (A) and invasion (B) capacity in the presence of either mock transfected, scramble siRNA, CrkI/II, CrkL siRNA or both CrkI/II and CrkL siRNA in combination. Image analysis of these assays was carried out using Scion Image software. A minimum of three experiments were performed. Error bars represent the standard error of the three experiments (A, B). Western blot analysis of proteins from whole cell lysates (MDA-231 1833TR) with an anti-CrkI/II or anti-CrkL sera was performed and actin protein levels were used as a loading control (C). All data that are statistically significant is illustrated as follows (* represents P < 0.05), ** represents P < 0.01, *** represents P < 0.001). [file bcr3183-S5.PDF]

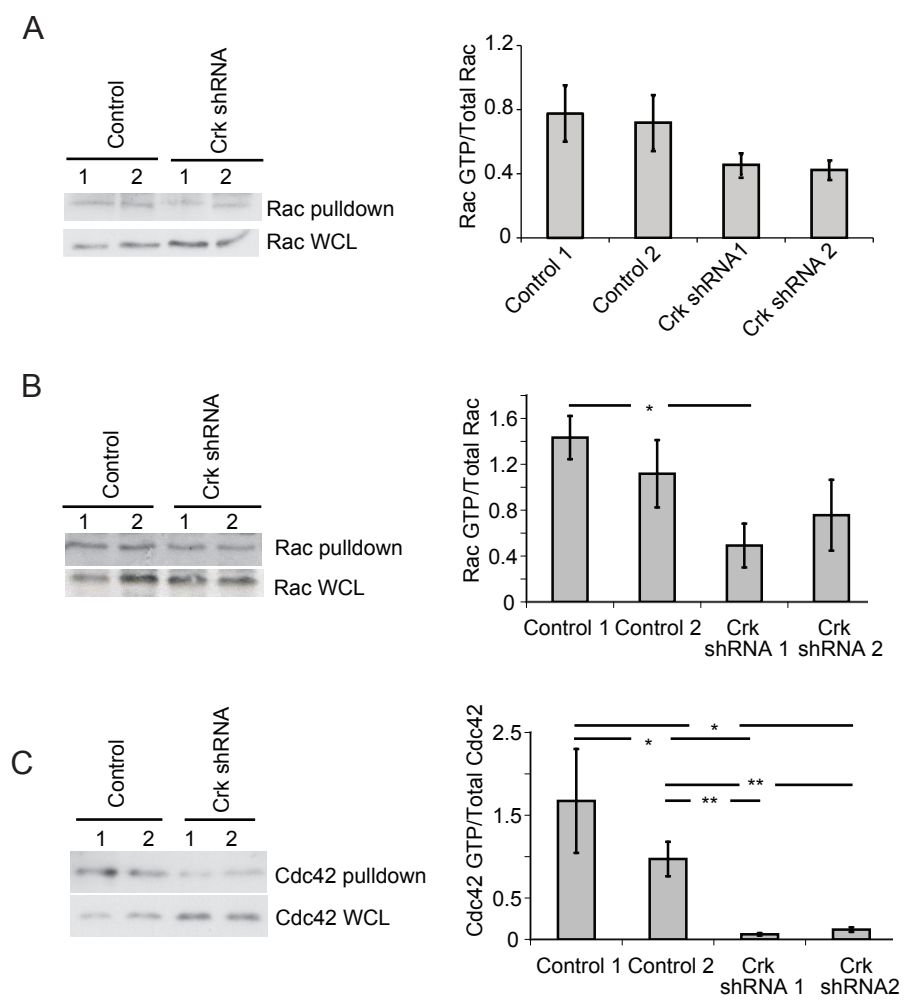

Supplement: Additional file 6 — Crk knockdown alters Cdc42 activation but does not significantly impact Rac activation. Rac1 pulldown experiments were performed after a four hour serum starvation, followed by lysis 30 minutes post-plating on collagen. All samples were analyzed at the same time, allowing for comparisons. The GTP bound form of endogenous Rac was precipitated by GST-CRIB and probed for anti-Rac1 sera. Total endogenous levels of Rac were visualized by immunoblot. Activated levels of Rac1 were quantified and compared to total Rac1 levels (n = 6) (A). In response to serum, Rac and Cdc42 activation was assessed utilizing GST-Pak-PBD or GST-WASP as binding partners for GTP loaded Rac and Cdc42 respectively. Pulldown experiments were performed after a four hour serum starvation, followed by 15 minutes serum stimulation. All samples were analyzed at the same time, allowing for comparisons. The GTP bound form of endogenous Rac was precipitated by GST-Pak-PBD and probed for anti-Rac1 sera. Total endogenous levels of Rac were visualized by immunoblot. Activated levels of Rac1 were quantified and compared to total Rac1 levels (n = 4) (B). The GTP bound form of endogenous Cdc42 was precipitated by GST-WASP and probed for anti-Cdc42 sera. Total levels of endogenous Cdc42 were visualized by immunoblot. Activated levels of Cdc42 were quantified as described above (C). Error bars represent SEM. All data that are statistically significant is illustrated as follows (* represents P < 0.05), ** represents P < 0.01, *** represents P < 0.001). [file bcr3183-S6.PDF]

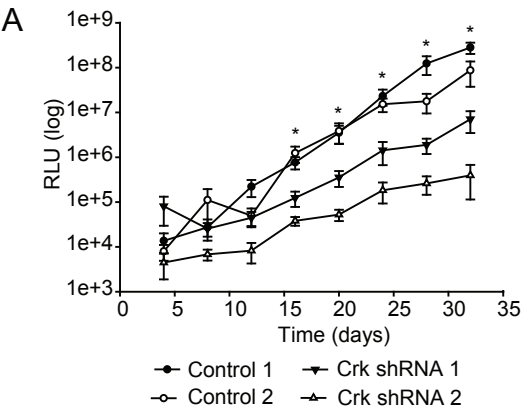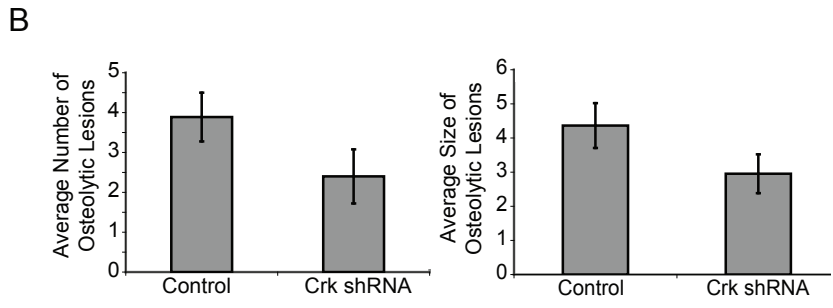

Supplement: Additional file 7 — Crk proteins are required for efficient outgrowth of breast cancer cells in the tibia. MDA-231 1833TR control cells or those expressing Crk shRNA were injected directly into the tibia and tumor outgrowth was measured by bioluminescence imaging. Tumor outgrowth within the tibia was quantified using limbs positive for bioluminescent activity, measured as relative luciferase units (RLU) over time and plotted on a log scale as the mean +/- SEM (A). The average number and size of osteolytic lesions from pooled control (n = 9) and Crk knockdown samples (n = 5) was quantified and plotted with SEM (B). All data that is statistically significant is illustrated as follows (* represents P < 0.05), ** represents P < 0.01, *** represents P < 0.001). [file bcr3183-S7.PDF]

A

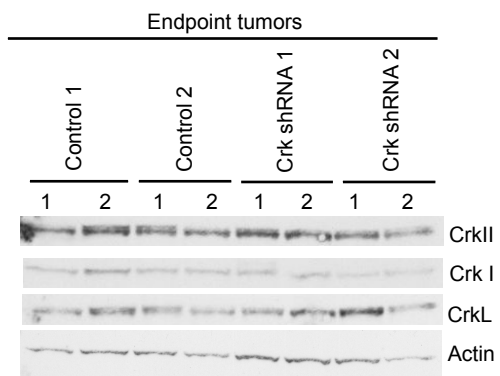

B

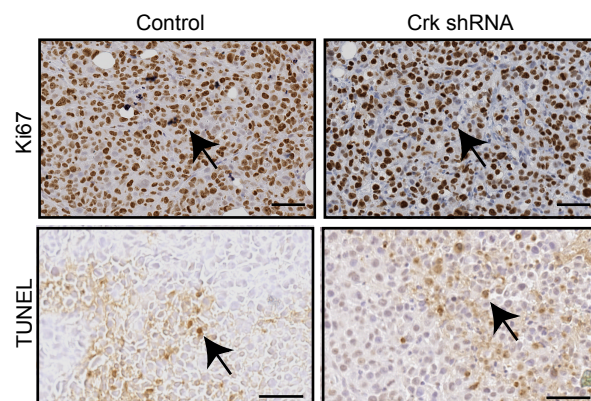

C

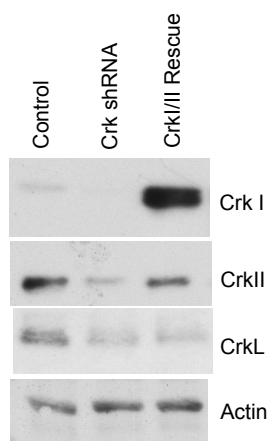

D

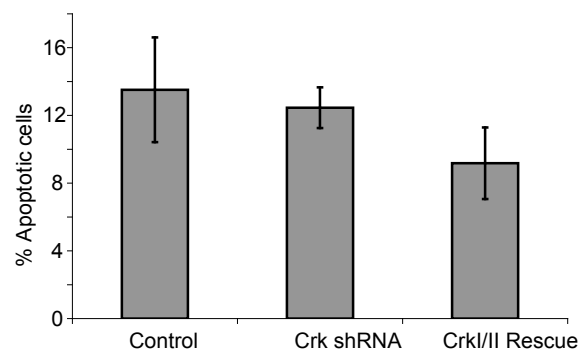

E

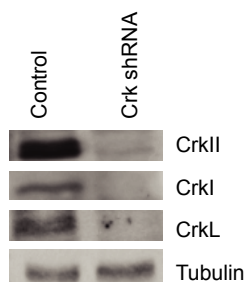

F

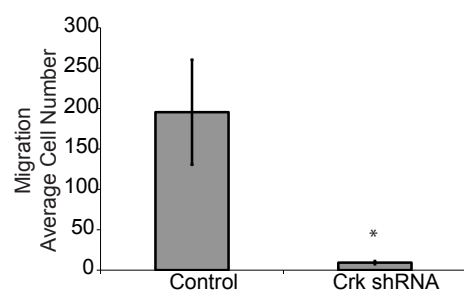

G

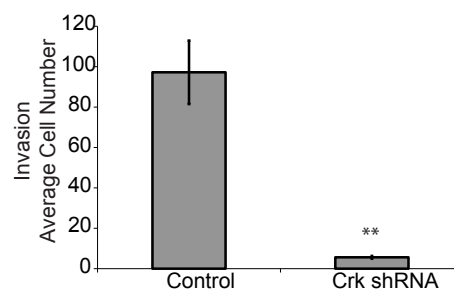

Supplement: Additional file 8 — Crk expression and growth kinetics in vitro and in vivo. Western blot analysis of Crk protein expression levels from tumor endpoint (A). Paraffin embedded sections of mammary tumors derived from 1833TR control and Crk shRNA cells were stained for Ki67 (positive staining represented by black arrows) and TUNEL (positive staining represented by black arrows). No significant differences were observed between control and Crk knockdown tumors for Ki67 and TUNEL. All images taken at 20x where the scale bar represents 50 μm. Arrows represent area of insert (B). CrkI/II rescue was examined via Western blot analysis of whole cell lysates (MDA-231 1833TR) with an anti-CrkI/II or anti-CrkL sera. Actin protein levels were used as a loading control (C). Paraffin embedded sections of mammary tumors derived from 1833TR control, Crk shRNA and CrkI/II rescue cells were stained for apoptosis via TUNEL staining and quantified using ImageScope (D). Crk knockdown of SUM1315 cells was examined via Western blot analysis of whole cell lysates with an anti-CrkI/II or anti-CrkL sera. Alpha-tubulin protein levels were used as a loading control (E). Control and Crk shRNA SUM1315 cells (8 × 10^4^ cells) were plated on transwells and were analyzed for migration (F) and invasion (G) towards 10% FBS 24 hours post-plating then quantified using ImageScope software (n = 3). All data that is statistically significant is illustrated as follows (* represents P < 0.05), ** represents P < 0.01, *** represents P < 0.001). [file bcr3183-S8.PDF]

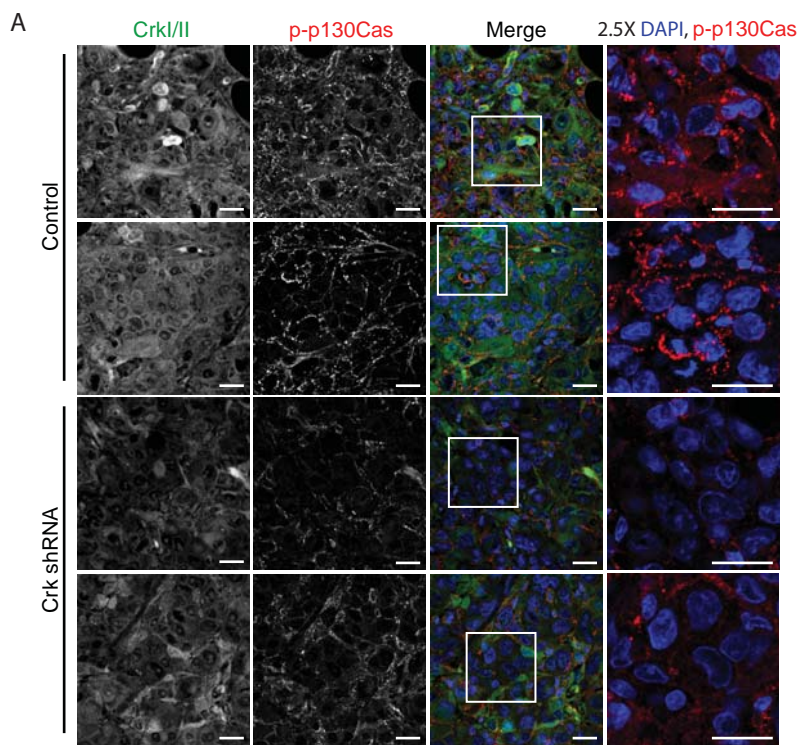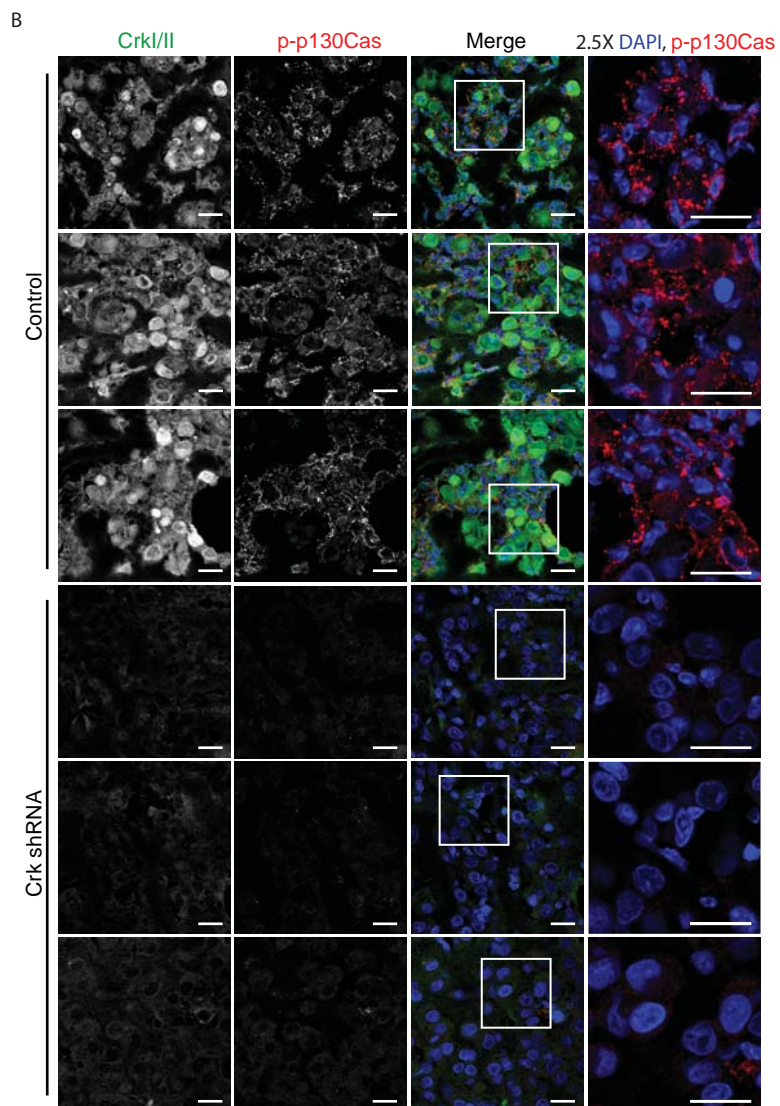

Supplement: Additional file 9 — Phosphorylated p130Cas is diminished in lesions derived from Crk knockdown cell lines. Immunofluorescence of CrkI/II and phosphorylated p130Cas was performed on paraffin embedded sections from control and Crk shRNA MDA-231 1833TR samples three days post-injection. Scale bars 20 μm (A). Immunofluorescence of CrkI/II and phosphorylated p130Cas was performed on paraffin embedded sections from control and Crk shRNA SUM1315 samples 3 days post-injection. Scale bars 20 μm (A). [file bcr3183-S9.PDF]

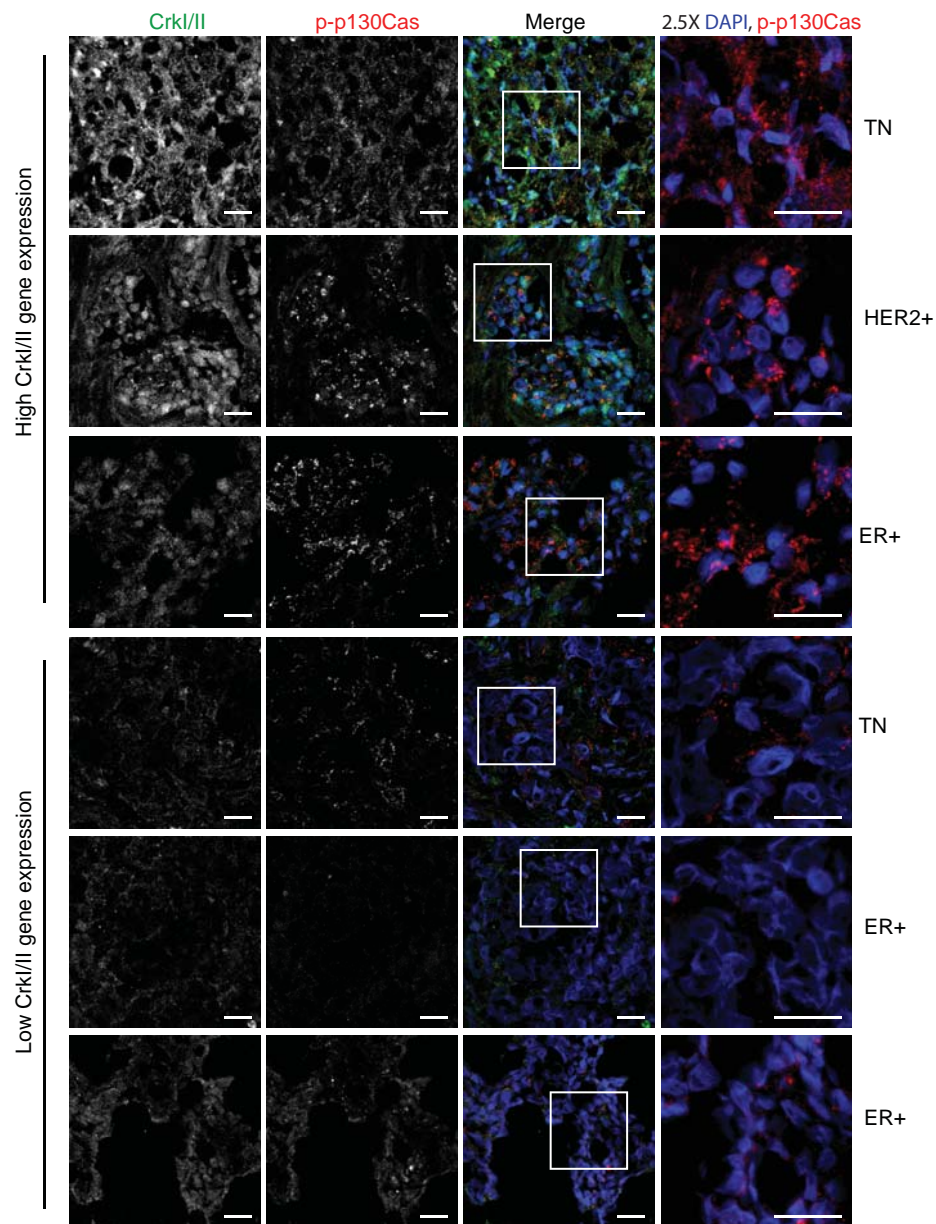

Supplement: Additional file 10 — Phosphorylated p130Cas expression correlates with CrkI/II expression in human breast cancer tumors. Immunofluoresence of CrkI/II and phosphorylated p130Cas was performed on frozen human basal breast cancer tissue. CrkI/II mRNA expression from these patients was also assessed by microarray analysis. TN = triple negative, ER = estrogen receptor. Scale bars 20 μm (A). [file bcr3183-S10.PDF]
